# Novel Metronidazole Conjugates as Antimicrobial Agents

**DOI:** 10.1002/ddr.70114

**Published:** 2025-06-10

**Authors:** Erol Akgün, Melike Demirayak, Leyla Yurttaş, Ülkiye Dudu Gül, Şeref Demirayak

**Affiliations:** ^1^ Department of Pharmaceutical Chemistry, Faculty of Pharmacy Marmara University İstanbul Türkiye; ^2^ Department of Pharmaceutical Chemistry, Institute of Graduate Education Anadolu University Eskişehir Türkiye; ^3^ İnci Dental Polyclinic Eskişehir Türkiye; ^4^ Department of Pharmaceutical Chemistry, Faculty of Pharmacy Anadolu University Eskişehir Türkiye; ^5^ Department of Bioengineering, Faculty of Engineering Bilecik Şeyh Edebali University Bilecik Türkiye; ^6^ Department of Pharmaceutical Chemistry, Faculty of Pharmacy İstanbul Okan University İstanbul Türkiye

**Keywords:** aerobic microorganisms, FMN, metronidazole, nitroimidazole

## Abstract

Metronidazole (MTZ) is one of the oldest and still used anti‐infective nitroimidazole group drug. Although it is effective against anaerobic bacteria, protozoa, and parasites in clinical settings, it lacks efficacy against aerobic microorganisms. Due to its efficient molecular structure and synthetic usability due to the alcohol group in its framework, medicinal chemists aimed to reach new more effective molecules such as MTZ‐hybrids. In this study, 2‐[(benzimidazole/benzoxazole/benzothiazol‐2‐yl)thio]‐*N*‐[2‐(2‐methyl‐5‐nitro‐1*H*‐imidazol‐1‐yl)ethyl]acetamide (**5a−5j**) derivatives were synthesized and their antimicrobial and antifungal effects on aerobic bacteria and *Candida* spp. were investigated. Notably, most of newly designed conjugates displayed higher potency than MTZ itself, especially against Gram‐positive strains. Furthermore, chlorinated heterocyclic moieties provided the strongest effects. Docking studies using *E. coli* nitroreductase (PDB: 1IDT) revealed potential interactions with the flavin mononucleotide (FMN) cofactor, suggesting that these hybrids may undergo nitro‐group reduction analogous to MTZ. Additionally, pharmacokinetic predictions indicated generally favorable profiles.

## 1 Introduction

Nitroimidazoles are an important group of drugs used against anaerobic bacteria, protozoa, and parasites (Al‐Sha'er et al. 2013). The first member of the group, azomycin, 2‐nitroimidazole, was first isolated from *Streptomyces eurocidicus* in 1953, and its antibacterial effects as well as its antiprotozoal activities were determined in the following years. Based on the activity of this small molecule, the Rhone‐Poulenc group discovered metronidazole (MTZ) and dimetridazole, which have antitrichomonal activity, as a result of the studies conducted on nitroimidazoles. Following the success of this molecule carrying 5‐nitroimidazole, many different 5‐nitroimidazole derivatives were synthesized in the following years and their chemotherapeutic activities were determined (Smithen and Hardy 1982).

MTZ or 1‐(2‐hydroxyethyl)‐2‐methyl‐5‐nitroimidazole is an antimicrobial agent that has been used clinically for more than 50 years to treat infections caused by anaerobic Gram‐positive and Gram‐negative bacteria and protozoa; however, its efficacy against aerobic microorganisms has not been observed. In particular, it is highly effective against Gram‐negative bacteria *B. fragilis*, Gram‐positive bacteria *C. difficile*, and Gram‐negative microaerophilic bacterium *H. pylori*. It is used in the treatment of protozoal diseases such as amebiasis, giardiasis, and trichomoniasis. Antifungal and tuberculostatic activities have also been reported in various studies (Löfmark et al. 2010; Żwawiak et al. 2019). In addition to antibacterial agents such as tinidazole, secnidazole and ornidazole, pretomanid and delamanid are drugs known for their antitubercular activity that are nitroimidazole in structure (Papadopoulou et al. 2017; Leitsch 2019).

MTZ is a prodrug that is activated inside the cell by passing through the bacterial cell wall by passive diffusion. The resistance to the drug occurs when the enzymatic systems that reduce it are inactivated, the drug is converted to nontoxic derivatives by nim genes, and the drug is prevented from entering or being excreted (Löfmark et al. 2010). There may be side effects and toxicity problems in the use of MTZ. In addition to neurotoxicity and genotoxicity, nausea, vomiting, damaged tongue, and mouth sores can be observed (Löfmark et al. 2010; Bertinaria et al. 2003; Patel et al. 2021; Anand and Sharma 1997; Rocha‐Garduño et al. 2020).

MTZ is a good starting material for synthetic use and is preferred in various synthetic reactions used by many medicinal chemists due to the alcohol group in its structure. As in our studies (Benkli et al. 2003; Demirayak et al. 1999; Demirayak and Kiraz 1993), numerous derivatives of conjugates with different bioactive molecules have been synthesized while preserving the pharmacophore group, and their antimicrobial activity has been reported (Rossi and Ciofalo 2020; Pelozo et al. 2021; Koos et al. 1994; Li et al. 2022; Al‐Masri et al. 2012).

Clinical applications of new hybrid molecules of old nitroimidazoles are important against resistant bacteria and parasites. Pharmacophores and various chemical structures can be hybridized to reach optimum molecules in terms of physicochemical aspects. If we evaluate MTZ hybridization, 5‐NO_2_ group is necessary for activating redox and formation of free radicals. Although the nitro substituent exhibits toxic properties such as mutagenicity and hepatotoxicity, it is a critical group in the structure of nitroimidazole, nitrothiazole, and nitrofurans, which are anti‐infective agents. Based on the active potential of MTZ, many derivatives are synthesized via *N*‐alkyl (Patel et al. 2021; Jarrad et al. 2018). In this study, we synthesized 2‐[(benzimidazole/benzoxazole/benzothiazol‐2‐yl)thio]‐*N*‐[2‐(2‐methyl‐5‐nitro‐1*H*‐imidazol‐1‐yl)ethyl]acetamide (**5a−5j**) derivatives by preserving the 2‐methyl‐5‐nitroimidazole pharmacophore and investigated their antimicrobial activity on aerobic bacteria and *Candida* species.

## 2 Results and Discussion

### 2.1 Chemistry

**Scheme 1:**
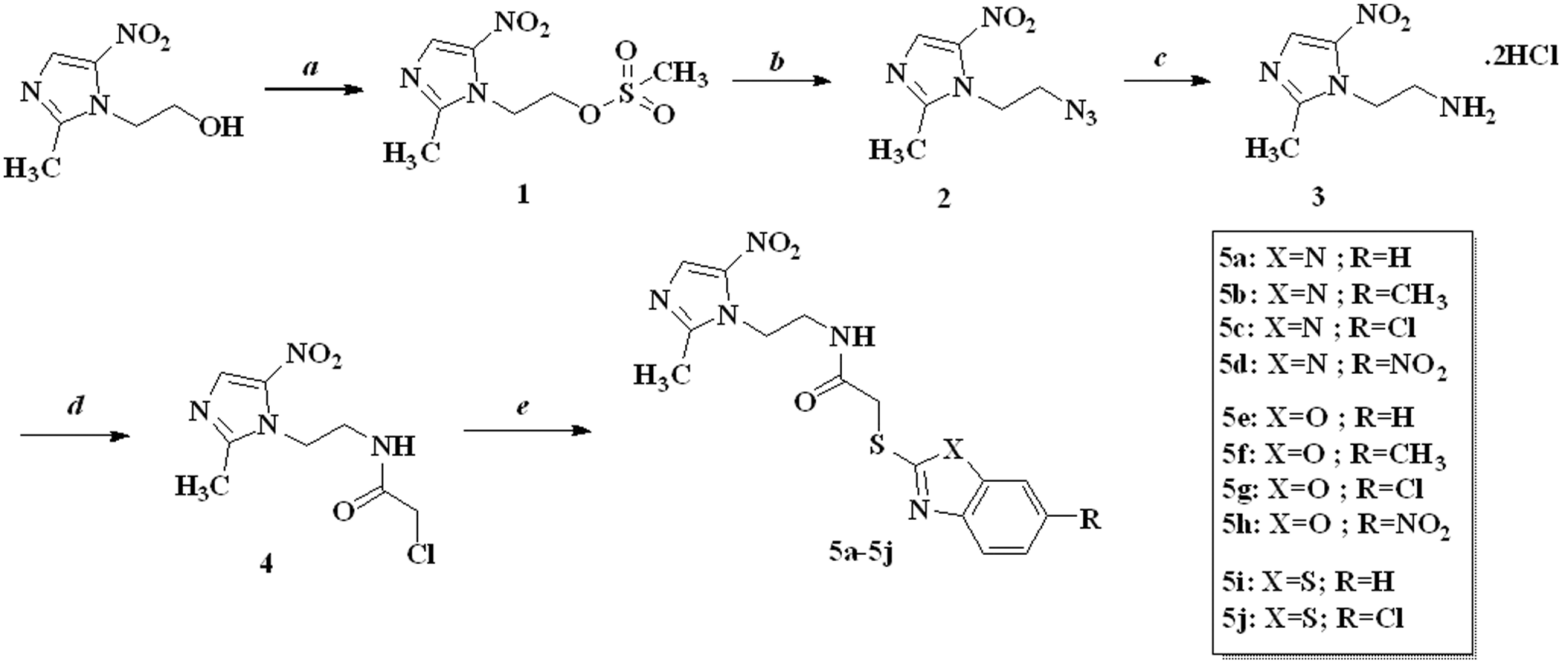
Synthesis of the compounds (**5a−3j**). Reaction conditions, reagents: (a) CH_3_SO_2_Cl, pyridine, 0°C–5°C; (b) NaN_3_, DMF, 70°C, 2 h; (c) Ph3P, anh. THF, 0°C−5°C, 3 h; then conc. HCl, refluxing, 5 h; (d) TEA, anh. THF, rt, 3 h; then ClCOCH_2_Cl, 0°C−5°C, overnight; (e) anh. THF, K_2_CO_3_, rt, overnight.

MTZ was used as a starting material in the synthesis of the compounds. 1‐(2‐hydroxyethyl)‐2‐methyl‐5‐nitroimidazole was first converted to amino by reaction with methanesulfonyl chloride, sodium azide, and triphenylphosphine, respectively. Then, 1‐(2‐aminoethyl)‐2‐methyl‐5‐nitroimidazole (**3**) was acetylated with chloroacetyl chloride to obtain the final products (**5a−5j**) with various 2‐mercaptoheteroaromatics as shown in Scheme 1. ^1^H‐NMR, ^13^C‐NMR, and MS spectra were taken to elucidate the structures of the resulting compounds. In the proton NMR spectra of the compounds, the amide N‐H present in each compound was observed between 8.52 and 10.75 ppm, NH‐CH_2_ between 4.33 and 4.64 ppm, N‐CH_2_ between 3.49 and 4.22 ppm and S‐CH_2_ between 4.10 and 3.97 ppm, CH_3_ on imidazole between 2.37 and 244 ppm. Other aromatic and aliphatic protons were observed at expected regions. In ^12^C‐NMR spectra of the compounds, common carbon signals belong to C=O, N‐CH_2_, NH‐CH_2_, S‐CH_2,_ and imidazole CH_3_ were observed in the range of 167.49−172.37 ppm, 45.47−43.67 ppm, 41.78−38.91 ppm, 36.75−32.79 ppm, 14.04−14.31 ppm, respectively. The mass spectrum of the compounds was taken in negative ion mode and was found to be consistent with the M‐1 peak.

### 2.2 Antimicrobial Activity

All of the final compounds, titled **5a−5j**, were tested on facultative anaerobic bacteria that cause intestinal infections and fungi. Two of the bacteria were Gram‐negative, four were Gram‐positive, and two were *Candida* species. MTZ and azithromycin were used as standard drugs for bacteria and voriconazole for fungi, and the results were presented in µM in Table 1. Minimum inhibitory concentrations (MIC) were found to be between 668.45 and 18.98 µM for the compounds, 730.99 and 182.75 µM for MTZ which is a drug used against anaerobic bacteria and protozoa, < 1.30 µM for azithromycin which is a wide spectrum antibiotic, and 11.17 µM for voriconazole against both *Candida* species.

**Table 1:** Antimicrobial activity of the compounds 5a−5j as MIC values (µM).

	A	B	C	D	E	F	G	H
**5a**	347.22	347.22	**173.61**	173.61	173.61	173.61	**86.81**	173.61
**5b**	334.22	167.11	**167.11**	167.11	668.45	**83.56**	**83.56**	167.11
**5c**	158.43	**79.21**	**19.80**	**79.21**	**79.21**	**19.80**	158.43	158.43
**5d**	154.32	**77.16**	308.64	**77.16**	**38.89**	**38.89**	154.32	308.64
**5e**	173.13	**86.57**	**86.57**	173.13	**43.25**	**86.57**	173.13	173.13
**5f**	166.67	**83.34**	**83.34**	**41.67**	**41.67**	**20.83**	166.67	333.33
**5g**	158.03	**39.51**	**79.01**	**39.51**	**39.51**	**19.75**	158.03	316.06
**5h**	153.94	**76.97**	**153.94**	**76.97**	**38.49**	**76.97**	153.94	307.88
**5i**	165.78	**41.45**	**82.89**	**82.89**	**41.45**	**82.89**	165.78	331.57
**5j**	151.88	**38.27**	**18.98**	**18.98**	**38.27**	151.88	151.88	303.77
S.D. 1	730.99	365.50	730.99	182.75	365.50	365.50	365.50	730.99
S.D. 2	< 1.30	< 1.30	< 1.30	< 1.30	< 1.30	< 1.30	—	—
S.D. 3	—	—	—	—	—	—	11.17	11.17

*Note:* Bold MIC values denote statistically significant activity (*p* < 0.05).

*Most active compounds. A: *E. coli* (ATCC 25922), B: *S. marcescens* (ATCC 8100), C: *S. aureus* (ATCC 29213), D: *S. epidermidis* (ATCC 12228), E: *E. faecalis* (ATCC 2942) F: *E. faecaum*, G: *C. albicans* (ATCC 24433), H: *C. krusei* (ATCC 6258) S.D. (Standard Drug): S.D. 1: Metronidazol; S.D. 2: Azytromycin; S.D. 3: Voriconazole, (A‐B: Gram− bacteria; C−F: Gram +bacteria; G‐H: Fungus)

*All microorganisms are facultative anaerobic organisms.

The synthesized compounds are MTZ hybrids and anaerobic bacteria were targeted. Antibacterial activity results were compared based on MTZ. When the effects of the compounds on Gram‐negative bacteria *E. coli* and *S. marcescens* were evaluated, it was seen that the compounds were effective against both and showed higher antimicrobial activity. While the MIC value of MTZ was 730.99 µM against *E. coli*, only two of the compounds showed activity at the half dose and the others at lower dose. While MTZ showed inhibition against *S. marcencens* at 365.50 µM, compound **5a** showed inhibition at about same concentration, other compounds inhibited at lower concentrations. Compound **5i** exhibited a MIC value of 41.45 µM, while compound **5j** exhibited a MIC value of 38.27 µM.

When the activities of the compounds against Gram‐negative bacteria were evaluated, it was seen that they were generally more active than Gram‐positive bacteria. All of the synthesized compounds showed the same or much higher activity against *S. aureus* than MTZ. While the MIC value of MTZ was 730.99 µM, the MIC value of **5e, 5f, 5g,** and **5i** was between 86.57 and 82.89 µM and the MIC value of **5c** and **5j** was 19.80 and 18.98 µM, respectively which were found to be excellent. These two compounds bear 5‐chlorobenzimidazole (**5c**) and 5‐chlorobenzothiazole (**5j**) rings. When the activity against *S. epidermidis* was examined, **5c, 5d, 5h,** and **5i** show the twice potency of MTZ (MIC: 182.75 µM), while **5f** and **5g** show four times the inhibition potential and **5j** shows 10 times the inhibition potential (MIC:18.95 µM). It was reported except **5b,** all compounds exhibited higher potency than MTZ (365.50 µM) against *E. faecalis*. Compounds **5d, 5e, 5f, 5g, 5h, 5i,** and **5j** had MIC values of 38.27−43.25 µM which shows 10 times higher antibacterial activity. The compounds were also found to have high activity against *E. faecium* and the activity potential was determined between 2 and 20 times higher. Compounds **5a** and **5j** had two times higher MTZ; compounds **5b, 5e, 5h,** and **5i** had four times higher; and **5c, 5d, 5f,** and **5g** had excellent MIC of 17.78−38.89 µM which was nearly 20 times higher. The compounds were found to have almost no antifungal effects. While voriconazole had a value of 11.17 µM, the compounds were found to exhibit the best MIC of 83.56 µM.

### 2.3 In Silico Studies

#### 2.3.1 Molecular Docking and MM/GBSA Studies

In a manner analogous to MTZ, newly synthesized compounds bearing a nitro‐substituted imidazole ring are hypothesized to undergo reduction into their active metabolites, thereby exerting therapeutic effects. To elucidate this mechanism, the interaction of *E. coli* nitroreductase—containing a flavin mononucleotide (FMN) cofactor—with the prodrug CB‐1954 (5‐(aziridin‐1‐yl)‐2,4‐dinitrobenzamide) was previously examined using the co‐crystal structure with PDB code 1IDT. This crystal structure revealed that the nitro group positioned close to the enzyme's cofactor, FMN, is reduced by the enzyme. Additionally, interactions involving hydrogen bonding with Thr41 and π−cation interactions with Phe124 were observed for this nitro group (Johansson et al. 2003).

**Figure 1:**
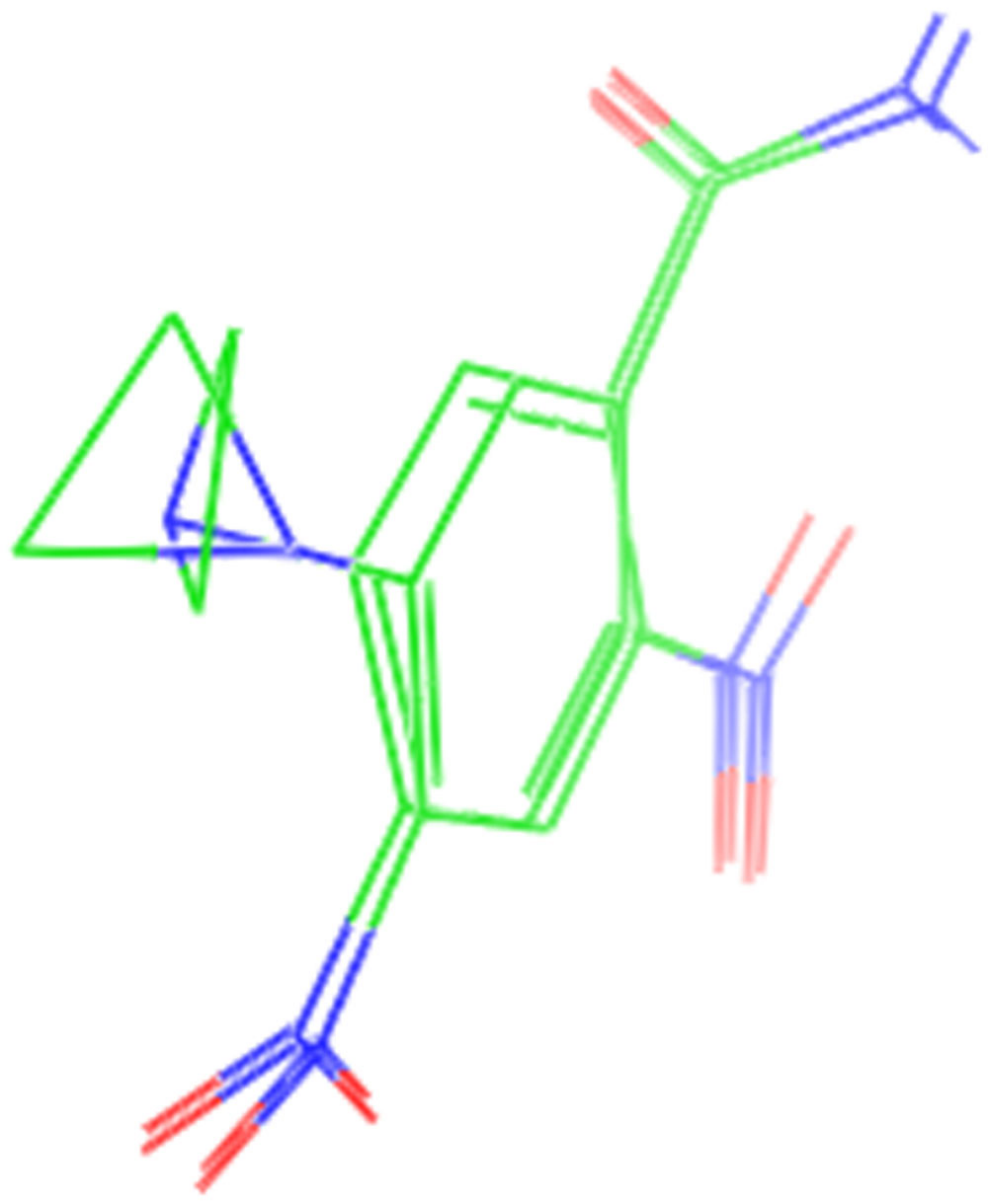
Superposition of the energy‐minimized structure of benzothiazole‐based inhibitor with the redocked ligand pose in the active site.

To validate the docking procedure at the active site, the co‐crystallized ligand was redocked, resulting in an RMSD value of 0.5782, indicating a reliable docking protocol (Figure 1).

Subsequently, the synthesized compounds were subjected to molecular docking analysis. Docking poses were evaluated, and preference was given to orientations where the compounds' imidazole‐NO₂ groups demonstrated the closest proximity to the FMN moiety. These selected poses underwent further evaluation using MM/GBSA analysis to examine their binding interactions, and the nature of the specific interactions formed by each compound was documented. Furthermore, the redocked pose of the reference compound CB‐1954 was analyzed comprehensively, employing both XP Gscore and MM/GBSA methodologies (Table 2).

**Table 2:** XP Gscore values and MM/GBSA binding interactions of synthesized compounds and compound CB‐1954.

Compounds	XP Gscore	MM/GBSA binding interactions
**5a**	−3.961	Lys14 (H‐bond) Asn117 (H‐bond) H_2_O (H‐bond) H_2_O (H‐bond) Thr41 (H‐bond) Phe124 (π−cation) Phe108 (π−π stacking)
**5b**	−4.313	Asn67 (H‐bond) Thr41 (H‐bond) Lys14 (H‐bond) H_2_O (H‐bond) Phe123 (π−π stacking) Phe123 (π−π stacking) Phe123 (π−cation)
**5c**	−4.175	Lys14 (H‐bond) Thr41 (H‐bond) Phe199 (π−π stacking) Phe199 (π−π stacking) Ala109 (halogen bond)
**5d**	−4.041	Lys14 (salt bridge) Lys119 (salt bridge) Asn67 (H‐bond) H_2_O (H‐bond) H_2_O (H‐bond) Lys14 (π−cation) Phe123 (π−cation) Phe70 (π−π stacking) Phe70 (π−π stacking)
**5e**	−2.595	Thr41 (H‐bond) Lys14 (H‐bond) H_2_O (H‐bond) Phe124 (π−cation)
**5f**	−3.069	Thr41 (H‐bond) Asn67 (H‐bond) H_2_O (H‐bond) Phe123 (π−π stacking))
**5g**	−4.036	Lys14 (H‐bond) Thr41 (H‐bond) H_2_O (H‐bond) Phe124 (π−cation)
**5h**	−4.765	Lys14 (H‐bond) Arg107 (H‐bond) H_2_O (H‐bond) H_2_O (H‐bond) Phe124 (π−cation)
**5i**	−4.199	Lys14 (H‐bond) Thr41 (H‐bond) Lys11 9 (H‐bond) H_2_O (H‐bond)
**5j**	−4.299	Lys14 (H‐bond) Thr41 (H‐bond) H_2_O (H‐bond) H_2_O (H‐bond) Phe124 (π−cation)
CB‐1954	−4.629	Thr41 (H‐bond) H_2_O (H‐bond) H_2_O (H‐bond) H_2_O (H‐bond) Phe124 (π−cation)

Although compound **5h** exhibited the lowest binding energy, its imidazole‐NO₂ group failed to form the Thr41 interaction, which was previously observed with the nitro group of the reference compound CB‐1954. The interactions established by the nitro groups attached to the imidazole ring in the docking poses of the compounds are detailed in Table 3.

**Table 3:** Interactions between residues and the nitro group attached to the imidazole ring of the compounds in poses obtained from MM/GBSA results.

Compounds	Interactions of imidazole‐linked NO₂ group with target residues
**5a**	Thr41 (H‐bond)
**5b**	Lys14 (H‐bond)
**5c**	Thr41 (H‐bond)
**5d**	—
**5e**	Thr41 (H‐bond)
**5f**	Thr41 (H‐bond)
**5g**	Thr41 (H‐bond), Phe124 (π−cation)
**5h**	Phe124 (π−cation)
**5i**	Lys14 (H‐bond)
**5j**	Thr41 (H‐bond), Phe124 (π−cation)
Ligand	Thr41 (H‐bond), Phe124 (π−cation)

**Figure 2:**
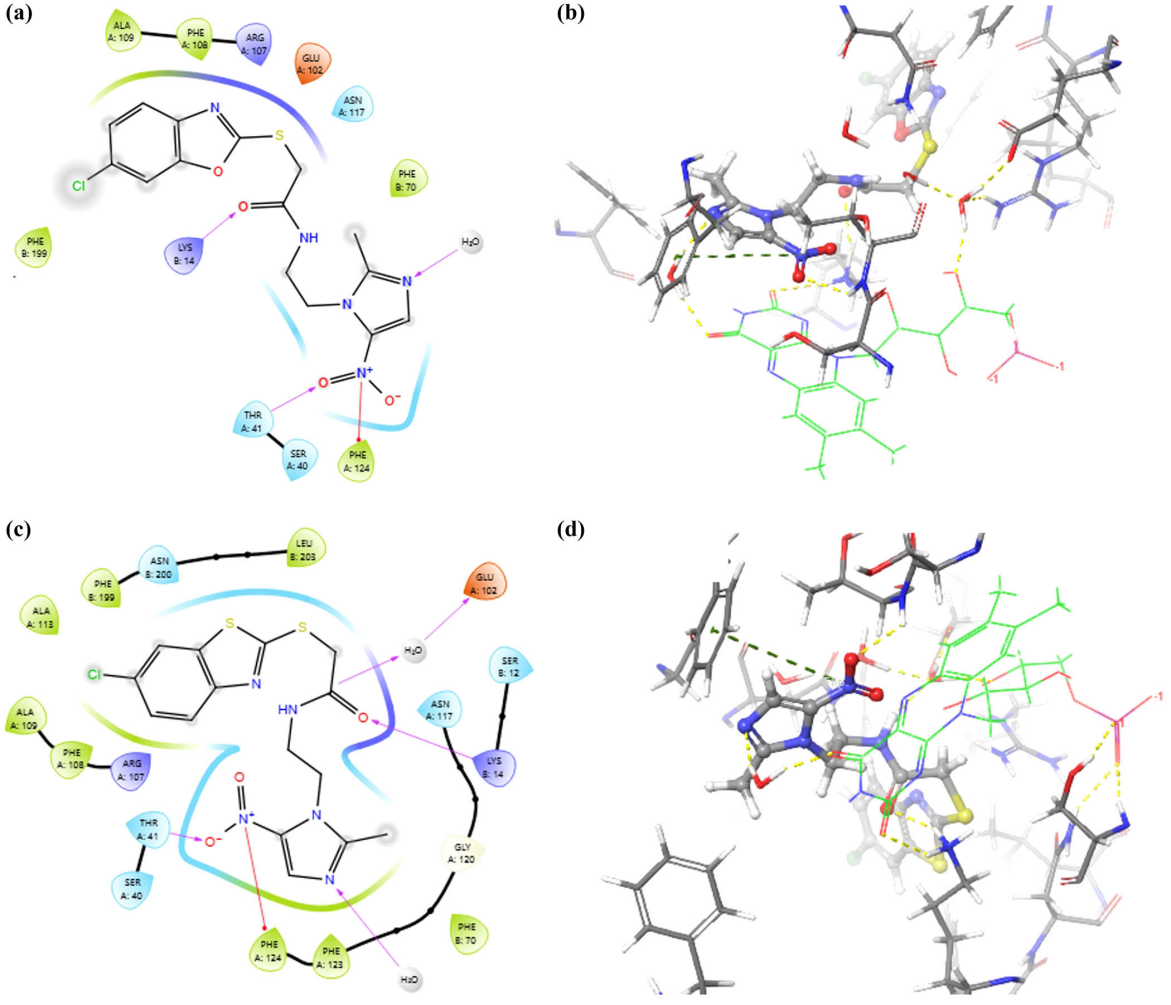
(a) Two‐dimensional (2D) representations illustrating interactions between **5g** and active site. (b) Three‐dimensional (3D) representations illustrating interactions between **5g** and active site. (c) Two‐dimensional (2D) representations illustrating interactions between **5i** and active site. (d) Three‐dimensional (3D) representations illustrating interactions between **5i** and active site.

Among the compounds, the imidazole‐linked NO₂ groups of compounds **5d** did not exhibit any interactions. Compounds **5b** and **5i** formed hydrogen bonds with Lys14; however, the distance between the NO₂ group and FMN in these poses was found to be large. Compound **5h** established a π−cation interaction with Phe124, whereas compounds **5a, 5c, 5e,** and **5f** formed only hydrogen bonds with Thr41. Both Thr41 hydrogen bonding and Phe124 π−cation interactions were simultaneously observed exclusively in compounds **5g** and **5j** (Figure 2).

#### 2.3.2 Pharmacokinetic Parameters Prediction

Pharmacokinetic parameters for the final compounds were predicted using the QikProp tool (Schrödinger Release 2024f). Before analysis, each compound was prepared using LigPrep. The synthesized compounds exhibited molecular weights ranging from 360.39 to 411.88 g/mol, all within acceptable limits for favorable oral bioavailability. LogP values of these compounds fell within ideal ranges, indicating balanced lipophilicity. Additionally, aqueous solubility (LogS) values were all within acceptable thresholds, suggesting sufficient solubility for oral administration. Most compounds showed high predicted human oral absorption with no violations of Lipinski's Rule of Five or Jorgensen's Rule of Three. However, compounds **5d** and **5h** demonstrated medium predicted oral absorption and each had one violation of both Lipinski's and Jorgensen's rules (Table 4).

**Table 4:** Some properties of synthesized compounds from QikProp.

	MW	HBA	HBD	Log P o/w	Log S for aqueous solubility	%Human oral absorbtion (±20%)	Qualitative model of human oral absorption	Lipinski rule of 5 violation	Jorgensen's rule of 3 violations
**5a**	360.39	6.5	2	1.750	−3.512	76	High	0	0
**5b**	374.41	6.5	2	2.111	−4.637	75	High	0	0
**5c**	394.83	6.5	2	2.198	−3.063	86	High	0	0
**5d**	405.39	7.5	2	1.123	−4.273	40	Medium	1	1
**5e**	361.37	7	1	1.769	−3.442	76	High	0	0
**5f**	375.40	7	1	2.081	−4.212	80	High	0	0
**5g**	395.82	7	1	2.257	−4.175	79	High	0	0
**5h**	406.37	8	1	1.064	−3.760	44	Medium	1	1
**5i**	377.44	6.5	1	2.525	−4.337	86	High	0	0
**5j**	411.88	6.5	1	2.774	−5.086	82	High	0	0

Abbreviations: HBA, H‐bond acceptor; HBD, H‐bond donor; MW, molecular weight.

## 3 Materials and Methods

### 3.1 Chemistry

The compounds were synthesized from commercially available solvents, starting materials and reagents (Merck, SigmaAldrich, Fluka). The melting points of the resulting compounds were determined with the Thermo Scientific melting point (Thermo Scientific, 9300, USA) device. The NMR spectra of the compounds were taken with a Bruker 300 Ultrashield spectrometer by dissolving them in DMSO‐*d*_6_, and the MS spectra were taken with G6125B LC/MSD.

#### 3.1.1 **Synthesis of 2‐(2‐Methyl‐5‐Nitro‐1** ***H*** **‐Imidazol‐1‐yl)Ethyl Methanesulfonate (1)**

MTZ is dissolved in pyridine and cooled to 0°C–5°C. Then, 1.2 equivalents of methanesulfonyl chloride is added dropwise. After the addition is complete, the reaction mixture is poured into water to precipitate the product, which is then recrystallized from chloroform (Demirayak and Kiraz 1993).

#### 3.1.2 **Synthesis of 1‐(2‐Azidoethyl)‐2‐Methyl‐5‐Nitro‐1** ***H*** **‐Imidazole (2)**

2‐(2‐Methyl‐5‐nitro‐1*H*‐imidazol‐1‐yl)ethyl methanesulfonate is dissolved in dimethylformamide (DMF), and 1.1 equivalents of sodium azide are added. The mixture is heated at 70°C for 2 h. Reaction completion is monitored by thin‐layer chromatography (TLC). The DMF is removed under reduced pressure, and the residue is extracted with ethyl acetate. After evaporation of the ethyl acetate, the remaining solid is recrystallized from an ethyl acetate–petroleum ether mixture (Demirayak and Kiraz 1993).

#### 3.1.3 **Synthesis of 2‐(2‐Methyl‐5‐Nitro‐1** ***H*** **‐Imidazol‐1‐yl)Ethylamine Dihydrochlorid (3)**

1‐(2‐Azidoethyl)‐2‐methyl‐5‐nitro‐1*H*‐imidazole is dissolved in anhydrous THF and cooled to 0°C–5°C. An equimolar amount of triphenylphosphine is then added dropwise. After stirring for 3 h, 100 mL of concentrated HCl is added, and the mixture is refluxed for 5 h. It is then evaporated to dryness, and the residue is extracted with water and ethyl acetate. The aqueous phase is evaporated, and the solid obtained is recrystallized from methanol (Demirayak and Kiraz 1993).

#### 3.1.4 **Synthesis of 2‐Chloro‐** ***N*** **‐(2‐(2‐Methyl‐5‐Nitro‐1** ***H*** **‐Imidazol‐1‐yl)Ethyl)Acetamide (4)**

2‐(2‐Methyl‐5‐nitro‐1*H*‐imidazol‐1‐yl)ethylamine dihydrochloride was slurried in anhydrous tetrahydrofuran (THF). Triethylamine (3.1 equivalents) was then added, and the mixture was stirred at room temperature for 3 h. The reaction mixture was subsequently cooled to 0°C–5°C, followed by the dropwise addition of chloroacetyl chloride. Stirring was continued overnight. Afterward, the mixture was filtered, and the filtrate was concentrated under reduced pressure to afford a crude solid. This material was purified by column chromatography using ethyl acetate:methanol:triethylamine (90:5:5) as the mobile phase, yielding 2‐chloro‐*N*‐(2‐(2‐methyl‐5‐nitro‐1*H*‐imidazol‐1‐yl)ethyl)acetamide.

#### 3.1.5 **Synthesis of 2‐[(Benzimidazole/Benzoxazole/Benzothiazol‐2‐yl)Thio]‐** ***N*** **‐(2‐(2‐Methyl‐5‐Nitro‐1** ***H*** **‐Imidazol‐1‐yl)Ethyl)Acetamide Derivatives (5a−5j)**

In anhydrous THF, 2‐chloro‐*N*‐(2‐(2‐methyl‐5‐nitro‐1*H*‐imidazol‐1‐yl)ethyl)acetamide is suspended with 1.1 equivalents of K₂CO₃. Then, derivatives of 2‐mercaptoheterocyclic aromatic compounds are added. Once the reaction is determined to be complete by thin‐layer chromatography, water is introduced into the mixture and the mixture is filtered. The resulting solid is dried, then purified via column chromatography using a ethyl acetate:hexane (50:50) as the mobile phase, yielding compounds **5a–5j**.

#### 3.1.6 **2‐Chloro‐** ***N*** **‐[2‐(2‐Methyl‐5‐Nitro‐1** ***H*** **‐Imidazol‐1‐yl)Ethyl]Acetamide (4)**

^1^H NMR (300 MHz, DMSO‐*d*_6_) δ 8.44 (t, *J* = 6.1 Hz, 1H, NH), 8.03 (s, 1H, Imidazole's H), 4.36 (t, *J* = 5.9 Hz, 2H, N‐CH_2_), 4.02 (s, 2H, Cl‐CH_2_), 3.50 (q, *J* = 6.0 Hz, 2H, HN‐CH_2_), 2.41 (s, 3H, CH_3_). ^13^C NMR (75 MHz, DMSO‐*d*_6_) δ 167.00 (C=O), 151.89 (Imidazole's C_2_), 138.99 (Imidazole's C_5_), 133.67 (Imidazole's C_4_), 45.62 (Cl‐CH_2_), 42.80 (N‐CH_2_), 38.82 (N‐CH_2_), 14.32 (CH_3_).

#### 3.1.7 **2‐[(Benzimidazol‐2‐yl)Thio]‐** ***N*** **‐[2‐(2‐Methyl‐5‐Nitro‐1** ***H*** **‐Imidazol‐1‐yl)Ethyl]Acetamide (5a)**

M.P.: 195°C (decomp). ^1^H NMR (300 MHz, DMSO‐*d*_6_) δ 12.59 (bs, 1H, Bim's N‐H), 8.54 (t, *J* = 6.0 Hz, 1H, O = C‐NH), 8.01 (s, 1H, Imidazole's H), 7.44 (dd, *J* = 3.1 Hz, 2H, Bim's H_4,7_), 7.12 (d, *J* = 3.1 Hz, 2H, Bim's H_5,6_), 4.33 (t, *J* = 5.8 Hz, 2H, N‐CH_2_), 3.99 (s, 2H, S‐CH_2_), 3.49 (q, *J* = 6 Hz, 2H, HN‐CH_2_), 2.37 (s, 3H, CH_3_). ^13^C NMR (75 MHz, DMSO‐*d*_6_) δ 168.40 (C=O), 151.92 (imidazole's, C_2_), 150.13, 138.93, 133.69, 121.89, 117.14, 110.60, 45.76 (N‐CH_2_), 38.93 (HN‐CH_2_), 35.20 (S‐CH_2_), 14.29 (CH_3_). MS (*m/z*) [M‐H]^+^ for C_15_H_16_N_6_O_3_S calculated 360.39, found: 359.2.

#### 3.1.8 **2‐[(5‐Methylbenzimidazol‐2‐yl)Thio]‐** ***N*** **‐[2‐(2‐Methyl‐5‐nitro‐1** ***H*** **‐imidazol‐1‐yl)ethyl]acetamide (5b)**

M.P.: 91°C (decomp). ^1^H NMR (300 MHz, DMSO‐*d*_6_) δ 12.46 (bs, 1H, Bim's N‐H), 8.54 (t, *J* = 6.2 Hz, 1H, O=C‐NH), 8.01 (s, 1H, Imidazole's H), 7.38‐7.17 (m, 2H, Bim's H_4,7_), 6.94 (d, *J* = 8.2 Hz, 1H, Bim's H_6_), 4.33 (t, *J* = 5.8 Hz, 2H, N‐CH_2_), 3.97 (s, 2H, S‐CH_2_), 3.49 (q, *J* = 6 Hz, 2H, HN‐CH_2_), 2.38 (s, 3H, CH_3_), 2.37 (s, 3H, CH_3_). ^13^C NMR (75 MHz, DMSO‐*d*_6_) δ 168.45 (C=O), 151.92 (Imidazole's C_2_), 144.26, 142.03, 138.93, 136.23, 133.68, 131.43, 123.03, 117.70, 117.35, 110.73, 45.76 (N‐CH_2_), 38.91 (HN‐CH_2_), 35.24 (S‐CH_2_), 21.67 (Bim's CH_3_), 14.28 (imidazole's CH_3_). MS (*m/z*) [M‐H]^+^ for C_16_H_18_N_6_O_3_S calculated 374.42, found: 373.2.

#### 3.1.9 **2‐[(5‐Chlorobenzimidazol‐2‐yl)Thio]‐** ***N*** **‐[2‐(2‐Methyl‐5‐Nitro‐1** ***H*** **‐Imidazol‐1‐yl)Ethyl]Acetamide (5c)**

M.P.: 203°C‐204°C. ^1^H NMR (300 MHz, DMSO‐*d*_6_) δ 12.79 (bs, 1H, Bim's N‐H), 8.52 (t, *J* = 6.1 Hz, 1H, O=C‐NH), 8.00 (s, 1H, Imidazole's H), 7.49 (d, *J* = 2.0 Hz, 1H, Bim's H_4_), 7.43 (d, *J* = 8.5 Hz, 1H, Bim's H_7_), 7.14 (dd, *J* = 8.5, 2.1 Hz, 1H, Bim's H_6_), 4.33 (t, *J* = 5.8 Hz, 2H, N‐CH_2_), 4.00 (s, 2H, S‐CH_2_), 3.49 (q, *J* = 5.8 Hz, 2H, HN‐CH_2_), 2.37 (s, 3H, CH_3_). ^13^C NMR (75 MHz, DMSO) δ 168.20 (C=O), 152.03, 151.91 (Imidazole's C_2_), 143.33, 138.93, 135.66, 133.67, 126.31, 122.03, 117.73, 110.32, 45.73 (N‐CH_2_), 38.94 (HN‐CH_2_), 35.16 (S‐CH_2_), 14.29 (CH_3_). MS (*m/z*) [M‐H]^+^ for C_15_H_15_ClN_6_O_3_S calculated 394.84, found: 393.1.

#### 3.1.10 **2‐[(5‐Nitrobenzimidazol‐2‐yl)Thio]‐** ***N*** **‐[2‐(2‐Methyl‐5‐Nitro‐1** ***H*** **‐Imidazol‐1‐yl)Ethyl]Acetamide (5d)**

M.P.: 175°C−177°C. ^1^H NMR (300 MHz, DMSO‐*d*_6_) δ 13.32 (bs, 1H, Bim's N‐H), 8.53 (t, *J* = 6.2 Hz, 1H, O=C‐NH), 8.31 (s, 1H, Bim's H_4_), 8.07 (d, *J* = 8.9 Hz, 1H, Bim's H_6_), 7.99 (s, 1H, Imidazole's H), 7.61 (d, *J* = 8.9 Hz, 1H, Bim's H_7_), 4.34 (t, *J* = 5.9 Hz, 2H, N‐CH_2_), 4.06 (s, 2H, S‐CH_2_), 3.51 (q, *J* = 6 Hz, 2H, HN‐CH_2_), 2.38 (s, 3H, CH_3_). ^13^C NMR (75 MHz, DMSO) δ 167.92 (C=O), 156.28, 151.91 (Imidazole's C_2_), 143.59, 142.63, 140.37, 138.93, 133.67, 118.00, 113.74, 110.89, 45.73 (N‐CH_2_), 38.96 (HN‐CH_2_), 35.16 (S‐CH_2_), 14.31 (CH_3_). MS (*m/z*) [M‐H]^+^ for C_15_H_15_N_7_O_5_S calculated 405.39, found: 404.0.

#### 3.1.11 **2‐[(Benzoxazol‐2‐yl)Thio]‐** ***N*** **‐[2‐(2‐Methyl‐5‐Nitro‐1** ***H*** **‐Imidazol‐1‐yl)Ethyl]Acetamide (5e)**

M.P.: 176°C (decomp). ^1^H NMR (300 MHz, DMSO‐*d*_6_) δ 8.88 (s, 1H, NH), 8.03 (s, 1H, Imidazole's H), 6.96 (td, *J* = 7.6, 1.6 Hz, 1H, Box's H_5_), 6.85 (dd, *J* = 8.1, 1.5 Hz, 1H, Box's H_4_), 6.77 (td, *J* = 7.4, 1.5 Hz, 1H, Box's H_6_), 6.64 (dd, *J* = 7.7, 1.7 Hz, 1H, Box's H_7_), 4.63 (dd, *J* = 5.5 Hz, 2H, N‐CH_2_), 4.22 (dd, *J* = 6.4, 4.6 Hz, 2H, HN‐CH_2_), 3.99 (s, 3H, CH_2_), 2.42 (s, 3H, CH_3_). ^13^C NMR (75 MHz, DMSO) δ 172.37 (C=O), 155.95 (Box's C_2_), 151.74 (Imidazole's C_2_), 148.93, 139.26, 135.48, 133.68, 125.85, 121.47, 119.67, 116.60, 43.74 (N‐CH_2_), 41.70 (HN‐CH_2_), 32.82 (S‐CH_2_), 14.04 (Imidazole's CH_3_). MS (*m/z*) [M‐H]^+^ for C_15_H_15_N_5_O_4_S calculated 361.38, found: 360.1.

#### 3.1.12 **2‐[(6‐Methylbenzoxazol‐2‐yl)Thio]‐** ***N*** **‐[2‐(2‐Methyl‐5‐Nitro‐1** ***H*** **‐Imidazol‐1‐yl)Ethyl]Acetamide (5f)**

M.P.: 209°C−211°C. ^1^H NMR (300 MHz, DMSO‐*d*_6_) δ 8.64 (bs, 1H, NH), 8.03 (s, 1H, Imidazole's H), 6.78‐6.71 (m, 2H, Box's H_4,5_), 6.46 (d, *J* = 1.7 Hz, 1H, H_7_), 4.62 (t, *J* = 5.8 Hz, 2H, N‐CH_2_), 4.21 (t, *J* = 6 Hz, 2H, HN‐CH_2_), 3.98 (s, 2H, S‐CH_2_), 2.42 (s, 3H, Imidazole's CH_3_), 2.19 (s, 3H, Box's CH_3_). ^13^C NMR (75 MHz, DMSO) δ 172.37 (C=O), 155.76 (Box's C_2_), 151.74 (Imidazole's C_2_), 146.52, 139.25, 135.19, 133.68, 128.21, 126.16, 121.86, 116.42, 43.72 (N‐CH_2_), 41.70 (HN‐CH_2_), 32.79 (S‐CH_2_), 20.69 (Box's CH_3_), 14.05 (Imidazole's CH_3_). MS (*m/z*) [M‐H]^+^ for C_16_H_17_N_5_O_4_S calculated 375.40, found: 374.2.

#### 3.1.13 **2‐[(6‐Chlorobenzoxazol‐2‐yl)Thio]‐** ***N*** **‐[2‐(2‐Methyl‐5‐Nitro‐1** ***H*** **‐Imidazol‐1‐yl)Ethyl]acetamide (5g)**

M.P.: 194°C−195°C. ^1^H NMR (300 MHz, DMSO‐*d*_6_) δ 9.27 (bs, 1H, NH), 8.02 (s, 1H, Imidazole's H), 6.99 (dd, *J* = 7.2, 2.7 Hz, 1H, Box's H_5_), 6.88 (d, *J* = 8.1 Hz, 1H, Box's H_4_), 6.65 (d, *J* = 2.4 Hz, 1H, Box's H_7_), 4.62 (t, *J* = 5.3 Hz, 2H, N‐CH_2_), 4.20 (t, *J* = 5.5 Hz, 2H, HN‐CH_2_), 4.03 (s, 2H, S‐CH_2_), 2.42 (s, 3H, CH_3_). ^13^C NMR (75 MHz, DMSO‐*d*_6_) δ 172.34 (C=O), 156.14 (Box's C_2_), 151.88 (Imidazole's C_2_), 148.25, 139.28, 137.00, 133.61, 125.25, 122.61, 121.23, 117.92, 43.74 (N‐CH_2_), 41.74 (HN‐CH_2_), 32.99 (S‐CH_2_), 14.05 (Imidazole's CH_3_). MS (*m/z*) [M‐H]^+^ for C_15_H_14_ClN_5_O_4_S calculated 395.82, found: 394.1.

#### 3.1.14 **2‐[(6‐Nitrobenzoxazol‐2‐yl)Thio]‐** ***N*** **‐[2‐(2‐Methyl‐5‐Nitro‐1** ***H*** **‐Imidazol‐1‐yl)Ethyl]Acetamide (5h)**

M.P.: 210°C (decomp). ^1^H NMR (300 MHz, DMSO‐*d*_6_) δ 10.75 (bs, 1H, NH), 8.01 (s, 1H, Imidazole's H), 7.94 (dd, *J* = 9.0, 2.9 Hz, 1H, Box's H_5_), 7.57 (d, *J* = 2.9 Hz, 1H, Box's H_7_), 7.04 (d, *J* = 9.0 Hz, 1H, Box's H_4_), 4.64 (t, *J* = 5.5 Hz, 2H, N‐CH_2_), 4.22 (t, *J* = 5.5 Hz, 2H, HN‐CH_2_), 4.08 (s, 2H, S‐CH_2_), 2.44 (s, 3H, CH_3_). ^13^C NMR (75 MHz, DMSO‐*d*_6_) δ 172.37 (C=O), 158.35, 156.15, 151.78 (Imidazole's C_2_), 139.86, 139.28, 135.86, 133.58, 122.36, 117.39, 116.63, 43.67 (N‐CH_2_), 41.78 (HN‐CH_2_), 33.14 (S‐CH_2_), 14.07 (Imidazole's CH_3_). MS (*m/z*) [M‐H]^+^ for C_15_H_14_N_6_O_6_S calculated 406.37, found: 405.0.

#### 3.1.15 **2‐[(Benzothiazol‐2‐yl)Thio]‐** ***N*** **‐[2‐(2‐Methyl‐5‐Nitro‐1** ***H*** **‐Imidazol‐1‐yl)Ethyl]Acetamide (5i)**

M.P.: 114°C−116°C. ^1^H NMR (300 MHz, DMSO‐*d*_6_) δ 8.66 (t, *J* = 6.0 Hz, 1H, NH), 8.05 (dd, *J* = 7.0, 1.2 Hz, 1H), 8.01 (s, 1H, Imidazole's H), 7.84 (dd, *J* = 9.8, 1.5 Hz, 1H), 7.48 (t, *J* = 7.6 Hz, 1H, Bty's H_5_), 7.37 (t, *J* = 7.6 Hz, 1H, Bty's H_6_), 4.34 (t, *J* = 6.0 Hz, 2H, N‐CH_2_), 4.10 (s, 2H, SCH_2_), 3.50 (q, *J* = 6.0 Hz, 2H, HN‐CH_2_), 2.39 (s, 3H, CH_3_). ^13^C NMR (150 MHz, DMSO‐*d*_6_) δ 167.49 (C=O), 166.46 (Bty's C_2_), 153.00 (Bty's C_3a_), 151.90 (Imidazole's C_2_), 138.96, 135.24, 133.68, 126.86, 124.99, 122.31, 121.60, 45.70 (N‐CH_2_), 39.00 (HN‐CH_2_), 36.70 (S‐CH_2_), 14.31 (Imidazole's CH_3_). MS (*m/z*) [M‐H]^+^ for C_15_H_15_N_5_O_3_S_2_ calculated 377.44, found: 376.2.

#### 3.1.16 **2‐[(6‐Chlorobenzothiazol‐2‐yl)Thio]‐** ***N*** **‐[2‐(2‐Methyl‐5‐Nitro‐1** ***H*** **‐Imidazol‐1‐yl)Ethyl]Acetamide (5j)**

M.P.: 149°C−152°C. ^1^H NMR (300 MHz, DMSO‐*d*_6_) δ 8.55 (t, *J* = 6.1 Hz, 1H, NH), 8.04 (d, *J* = 8.6 Hz, 1H, Bty's H_4_), 8.00 (s, 1H, Imidazole's H), 7.88 (d, *J* = 2.1 Hz, 1H, Bty's H_7_), 7.41 (dd, *J* = 8.6, 2.1 Hz, 1H, Bty's H_5_), 4.34 (t, *J* = 5.9 Hz, 2H, N‐CH_2_), 4.09 (s, 2H, SCH_2_), 3.51 (q, *J* = 6.0 Hz, 2H, HN‐CH_2_), 2.38 (s, 3H, CH_3_). ^13^C NMR (75 MHz, DMSO‐*d*_6_) δ 169.40 (C=O), 167.34 (Bty's C_2_), 153.86 (Bty's C_3a_), 151.89 (Imidazole's C_2_), 138.94, 134.02, 133.66, 131.65, 124.95, 123.69, 121.03, 45.67 (N‐CH_2_), 38.98 (HN‐CH_2_), 36.75 (S‐CH_2_), 14.31 (CH_3_). MS (*m/z*) [M‐H]^+^ for C_15_H_14_ClN_5_O_3_S_2_ calculated 411.89, found: 410.1.

### 3.2 Antimicrobial Activity

The antimicrobial activities of the compounds were tested on various facultative anaerobic bacteria and fungi species. Two Gram‐negative bacteria, *E. coli* (ATCC 25922), *S. marcescens* (ATCC 8100); four Gram‐positive bacteria, *S. aureus* (ATCC 29213), *S. epidermidis* (ATCC 12228), *E. faecalis* (ATCC 2942), *E. faecaum*, and two *Canida* species, *C. albicans* (ATCC 24433), *C. krusei* (ATCC 6258) were used. MTZ and azithromycin were used as standard drugs against bacteria; voriconazole was used against *Candida*. The antimicrobial activity was determined by the Broth Microdilution technique as described in CLSI. Bacterial cultures were prepared in Mueller‐Hinton broth. RPMI was used as the medium for Candida species. Test compounds were dissolved in a 2% DMSO solution and studied in the range of 3.90−500 µM. After incubating the compounds at different concentrations at 37°C, resazurin was added and incubated for another 2 h. Then, fluorometric reading was performed to determine MIC values. The experiments were repeated twice for each compound and concentration (Evren et al. 2025).

### 3.3 In Silico Studies

#### 3.3.1 Molecular Docking and MM/GBSA Calculations

The binding modes of the synthesized compounds to *E. coli* nitroreductase enzyme (NTR) possessing FMN as a cofactor, co‐crystallized with the compound CB‐1954 (5‐(aziridin‐1‐yl)‐2,4‐dinitrobenzamide), were investigated using Schrödinger software (Schrödinger LLC, New York, NY, 2024). The crystal structure of the enzyme (PDB code: 1IDT) was retrieved from the RCSB Protein Data Bank (www.rcsb.org).

Ligand preparation was performed using LigPrep at pH 7.4  ± 2.0 (Schrödinger Release 2024a), and enzyme preparation was done using the Protein Preparation Wizard (Schrödinger Release 2024b). During protein preparation, standard preprocessing was carried out, protonation states were assigned with PROPKA at pH 7.0, and water molecules forming fewer than two hydrogen bonds with non‐water atoms were removed. A restrained minimization was then applied, converging heavy‐atom positions to an RMSD of 0.30 Å. Receptor grids were generated in Glide (Schrödinger Release 2024c), centered on the binding site of the co‐crystallized ligand and the FMN cofactor. Docking simulations were performed under extra precision (XP) mode with expanded sampling, and up to 50 poses were recorded for receptor ligands and final compounds selecting ligand poses based on the optimal proximity of the nitro group attached to the imidazole ring to the FMN cofactor.

MM/GBSA calculations were carried out using the Prime (Schrödinger Release 2024d) module of the Schrödinger Suite. The receptor structure was derived directly from the docking complex after the removal of the docked ligand, and ligand structures were taken from the previously selected docking poses exhibiting optimal proximity to the FMN cofactor.

Calculations utilized the VSGB solvation model and the OPLS4 force field, with input ligand partial charges enabled. Protein flexibility was incorporated by allowing residue flexibility within a 5.0 Å distance from the ligand. Minimization was employed as the sampling method, without constraints on flexible residues. Finally, the resulting complexes were analyzed to investigate interactions between the compounds or the co‐crystal ligand and the target protein. Docking scores for these selected poses were recorded accordingly. Maestro interface (Schrödinger Release 2024e) was employed for visualization of the obtained results.

#### 3.3.2 Pharmacokinetic Parameters Prediction

Pharmacokinetic parameters for the final compounds were predicted using the QikProp tool. Before analysis, each compound was prepared using LigPrep.

## 4 Conclusion

As a result, 10 new 2‐[(benzimidazole/benzoxazole/benzothiazol‐2‐yl)thio]‐*N*‐[2‐(2‐methyl‐5‐nitro‐1*H*‐imidazol‐1‐yl)ethyl]acetamide derivatives were successfully synthesized, and their structures were characterized by NMR and MS data. These compounds were then evaluated for their antimicrobial efficacy against a panel of facultative anaerobic Gram‐negative and Gram‐positive bacteria, as well as *Candida* species. Most of the derivatives displayed higher antibacterial activity than MTZ, particularly against Gram‐positive strains. Notably, the chlorinated heterocyclic moieties tended to yield the highest antimicrobial activities on average. Docking studies using *E. coli* nitroreductase (PDB: 1IDT) provided insights into possible binding interactions with FMN, introducing a hypothesis that these hybrids may be activated via the nitro‐group reduction pathway in anaerobic microorganisms. In addition, the pharmacokinetic predictions for the newly synthesized compounds generally indicated favorable profiles.

## Conflicts of Interest

The authors declare no conflicts of interest.

## Supporting information

Supplementary data.

## Data Availability

The data that supports the findings of this study are available in the Supporting Information material of this article.
