# Supplementary material for: Novel Metronidazole Conjugates as Antimicrobial Agents
Source: Drug Dev Res. 2025 Jun 10;86(4):e70114. doi: 10.1002/ddr.70114 (PMC12150256; doi:10.1002/ddr.70114)
Supplement: Supplementary file 1 — Supplementary data. [file DDR-86-e70114-s001.docx]

**Supplementary data**


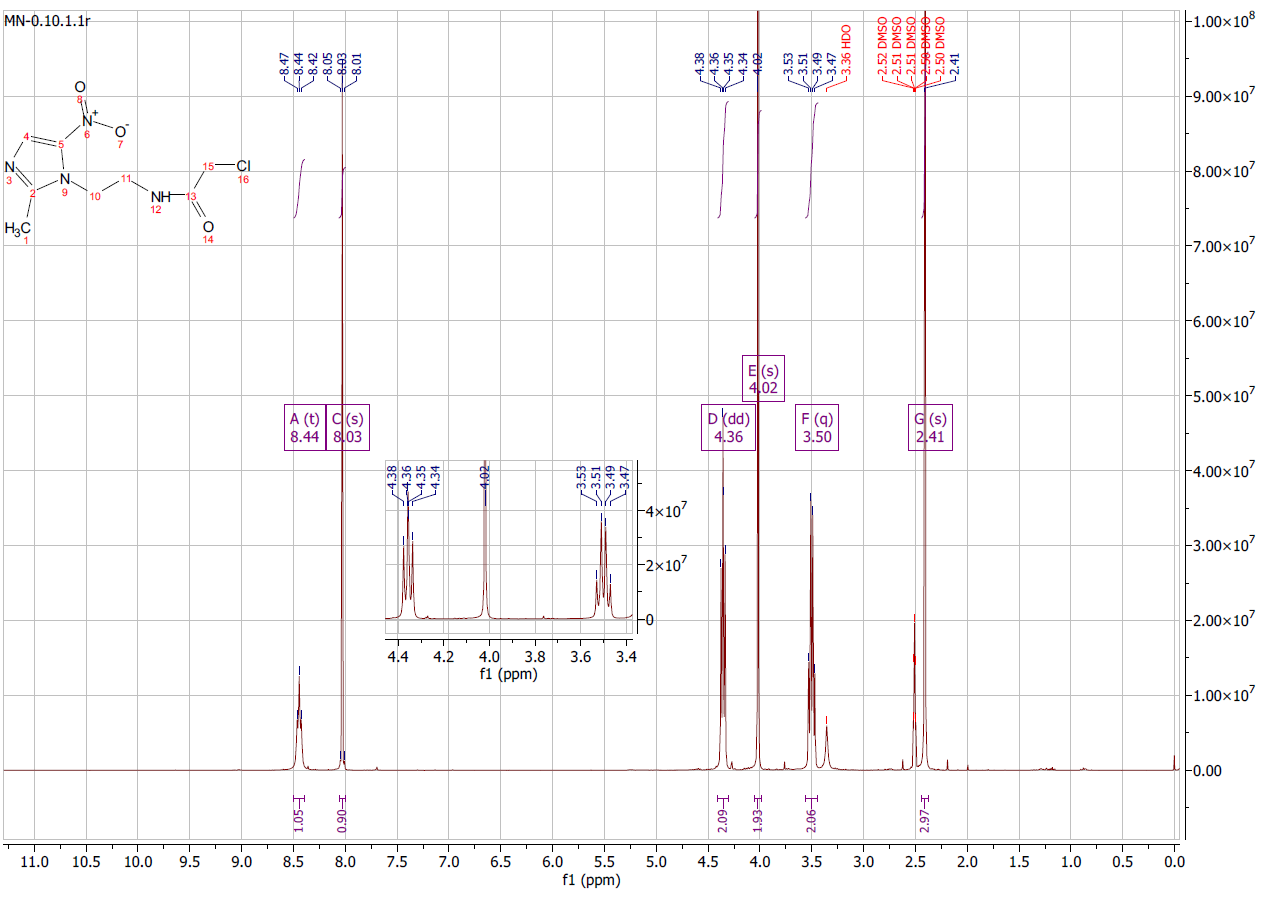


^1^H-NMR spectrum of the compound **4**


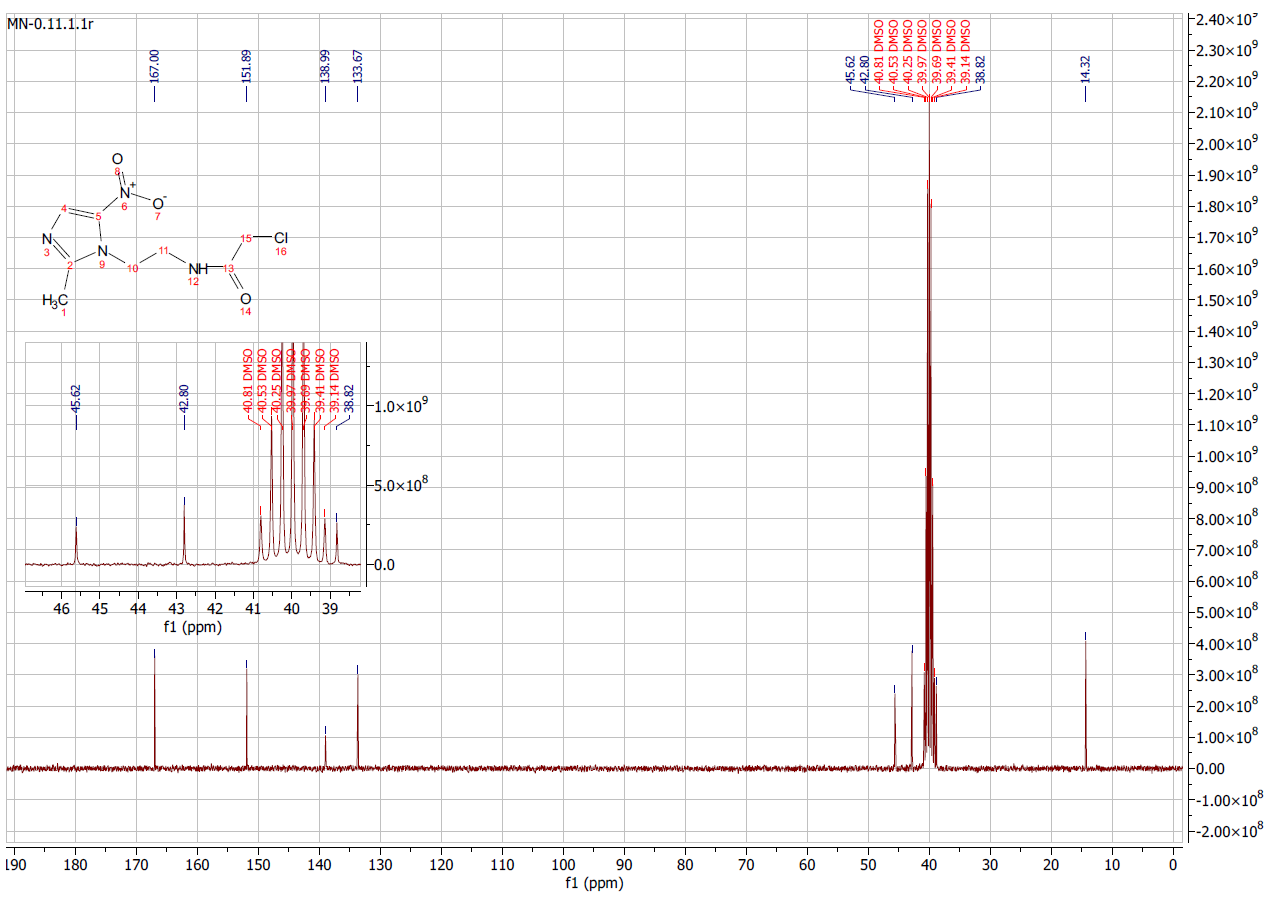


^13^C-NMR of the compound **4**


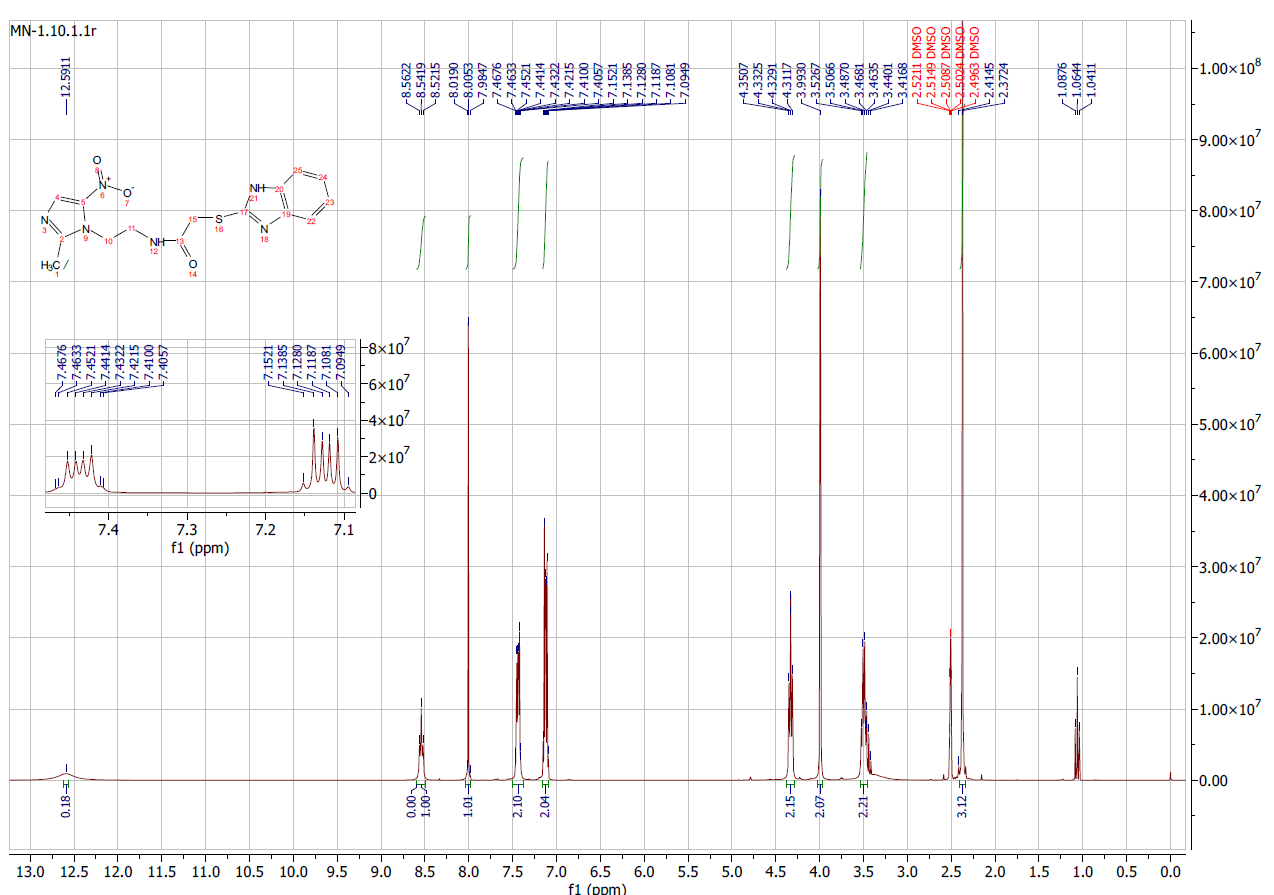


^1^H-NMR spectrum of the compound **5a**


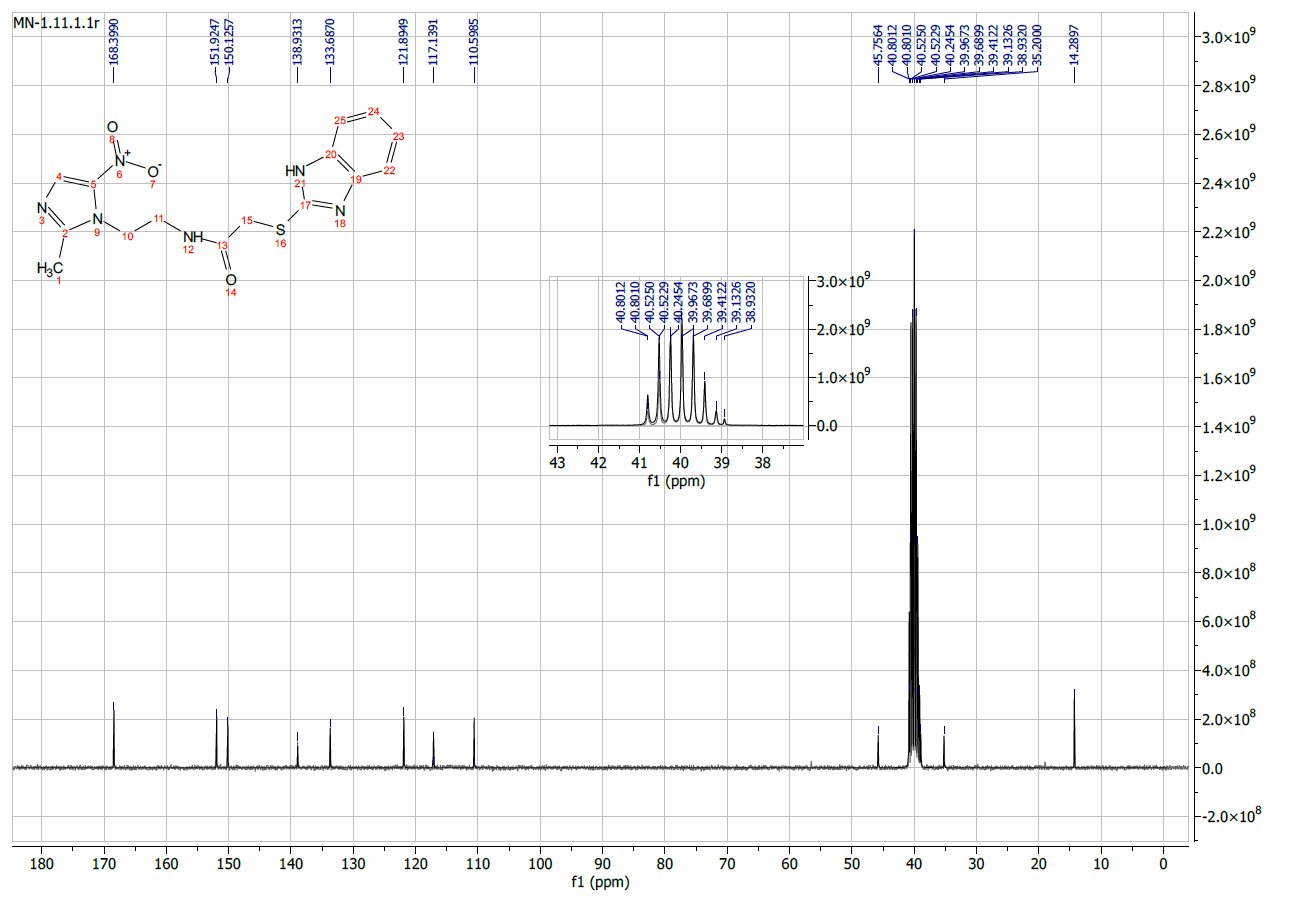


^13^C-NMR spectrum of compound **5a**


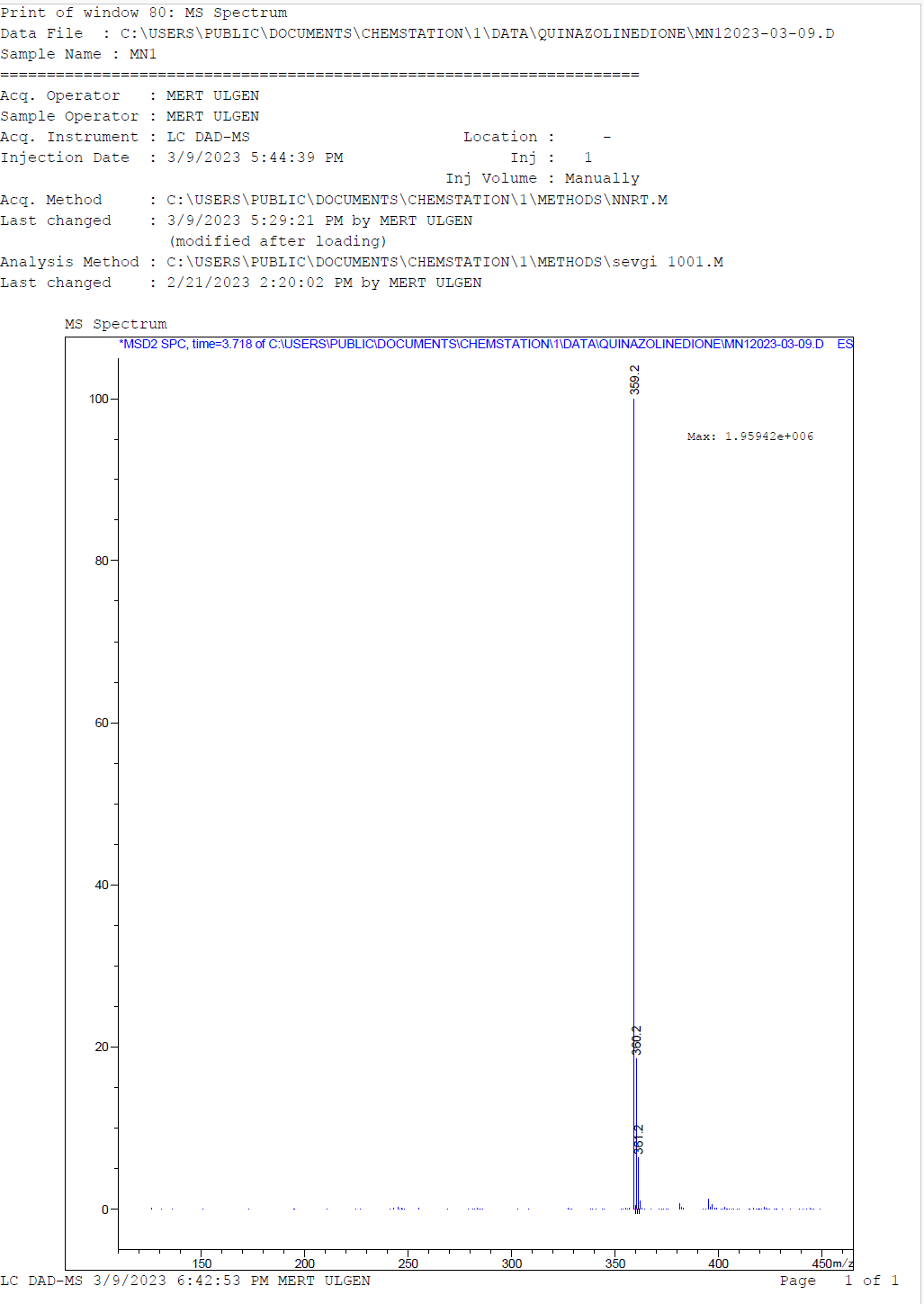


MS spectrum of the compound **5a**


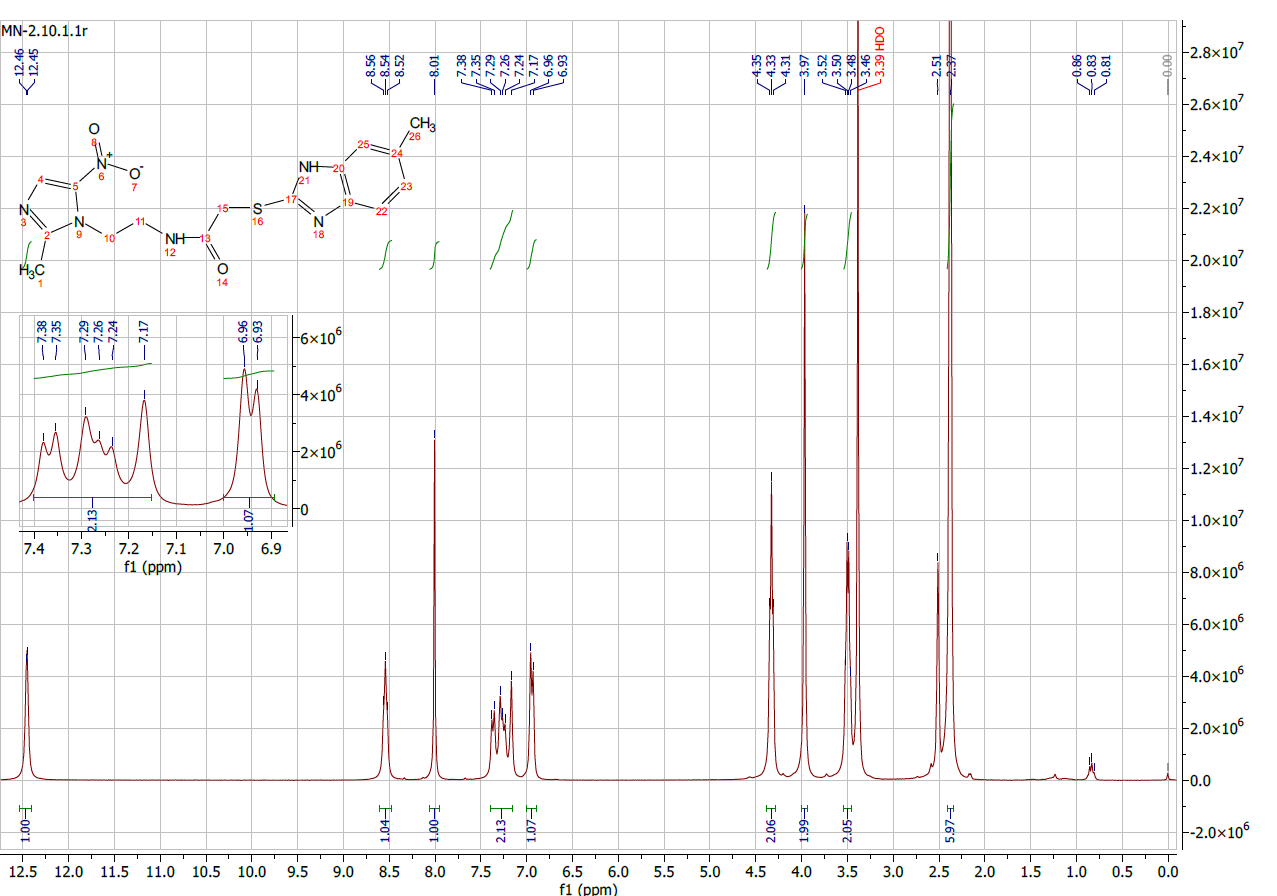


^1^H-NMR spectrum of the compound **5b**


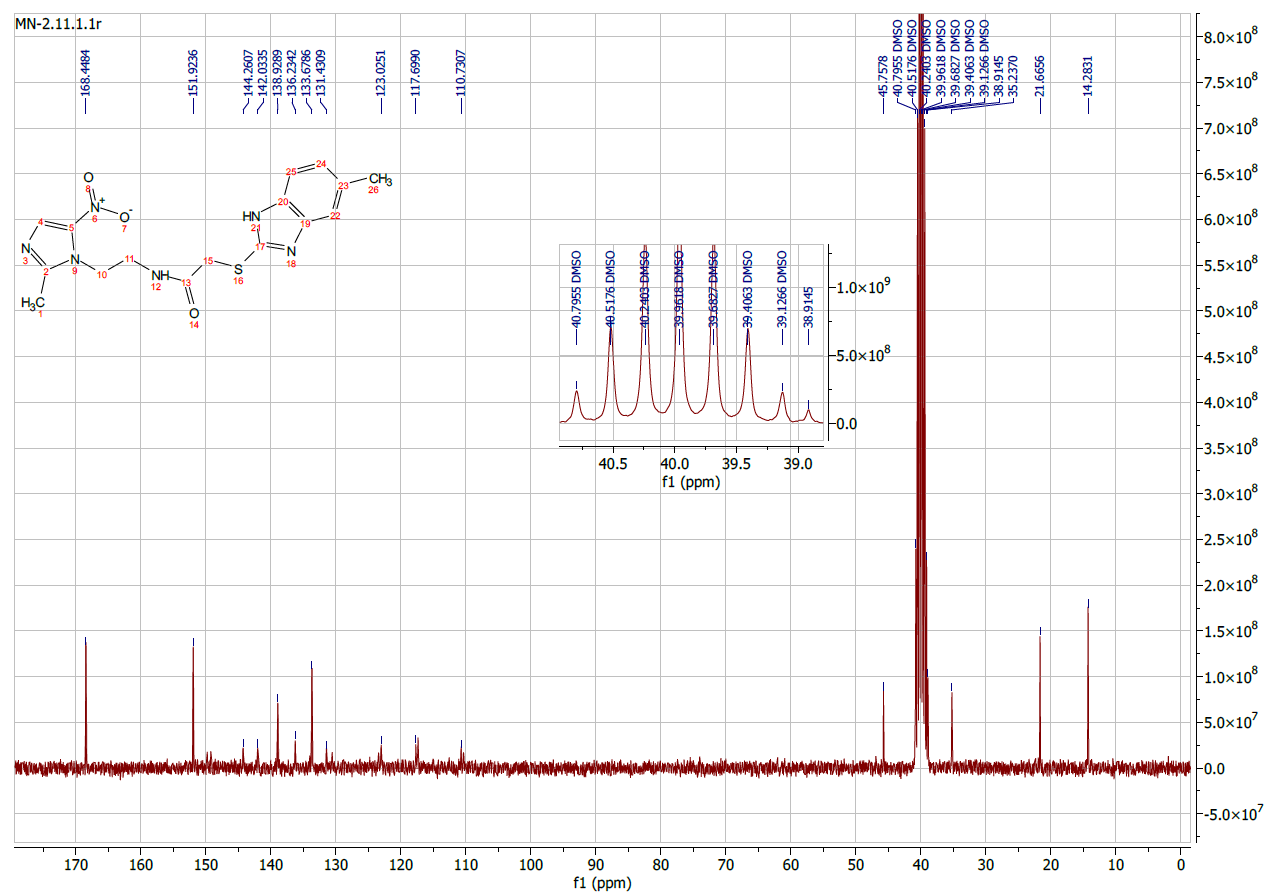


^13^C-NMR spectrum of compound **5b**


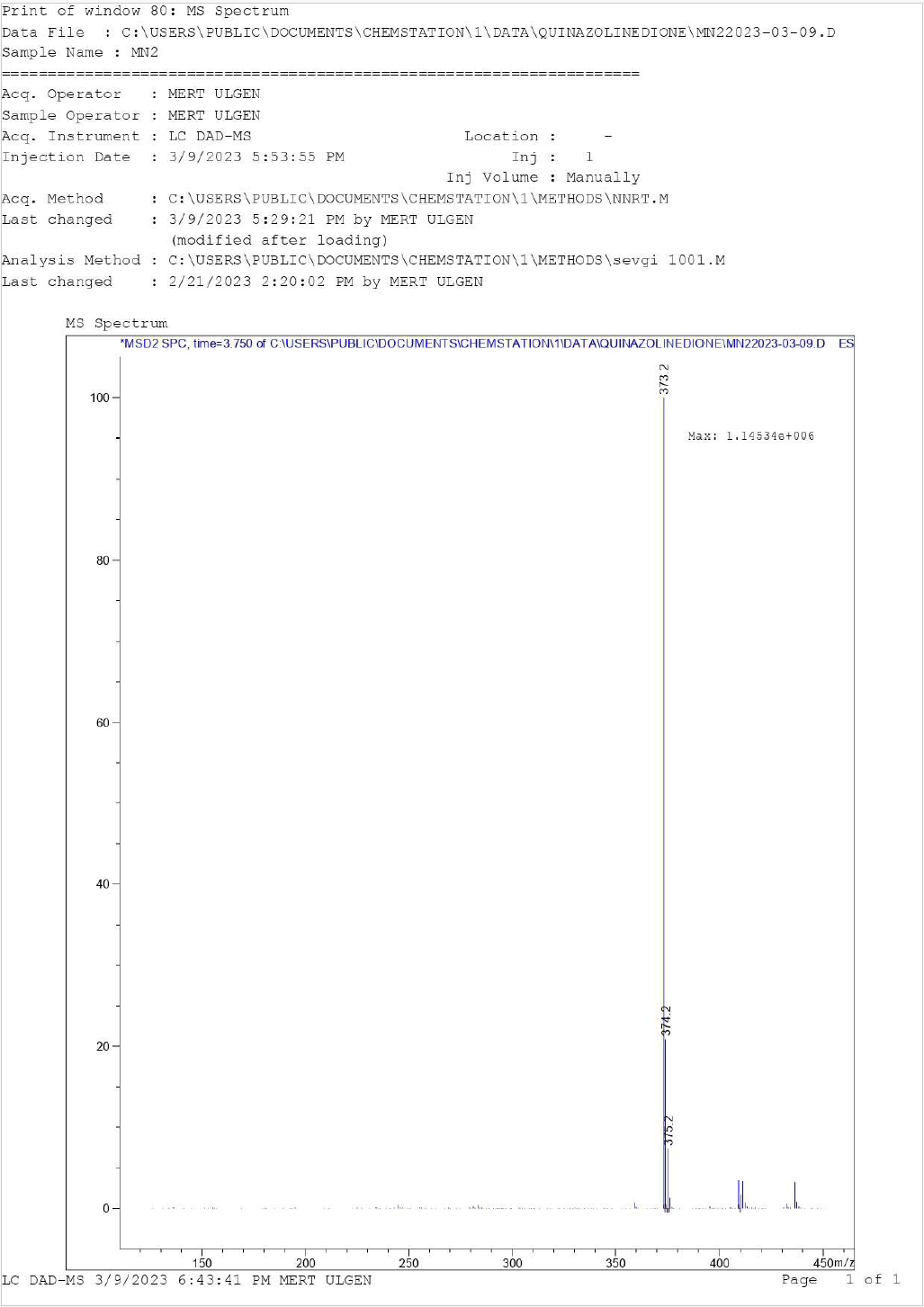


MS spectrum of the compound **5b**


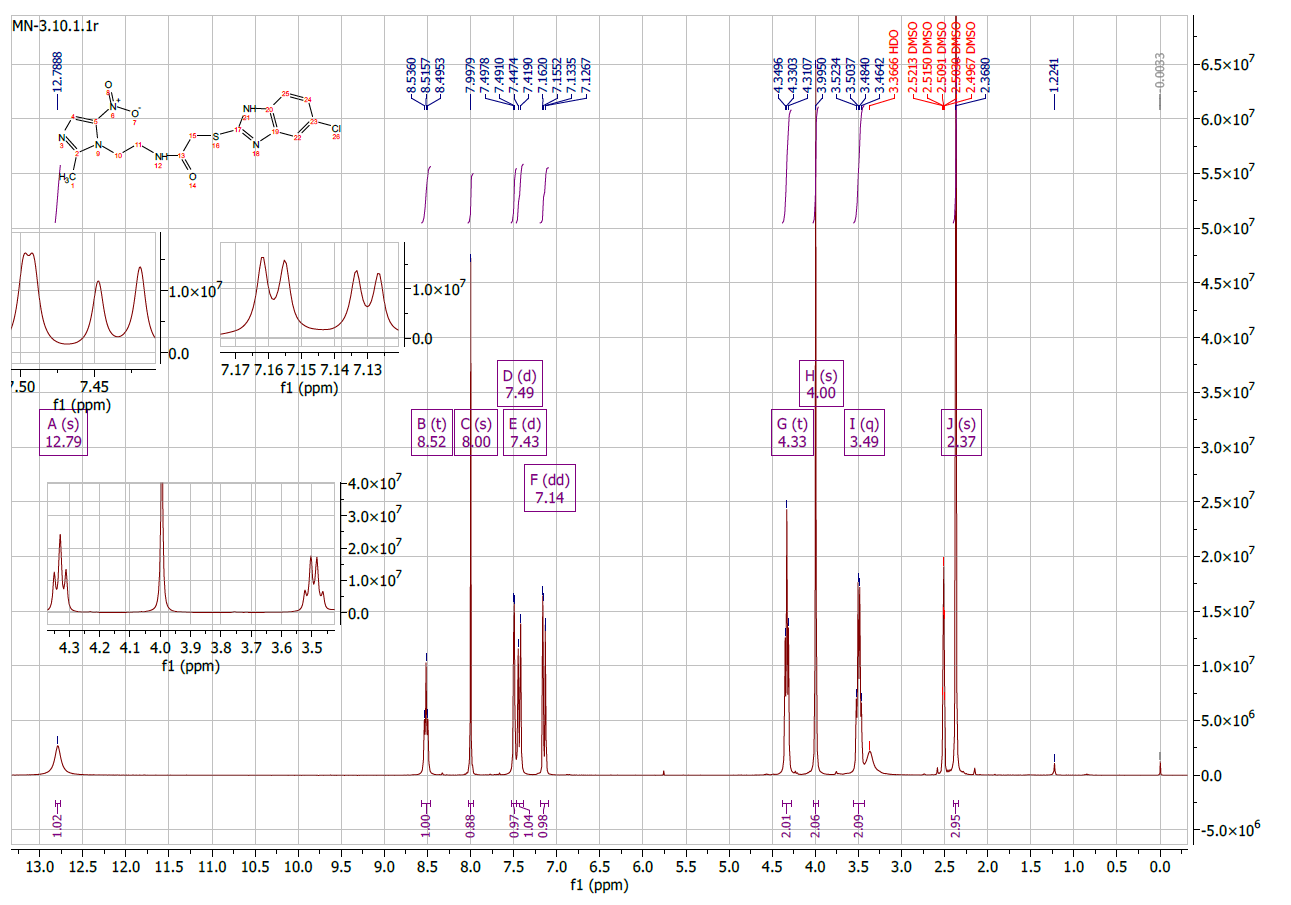


^1^H-NMR spectrum of the compound **5c**


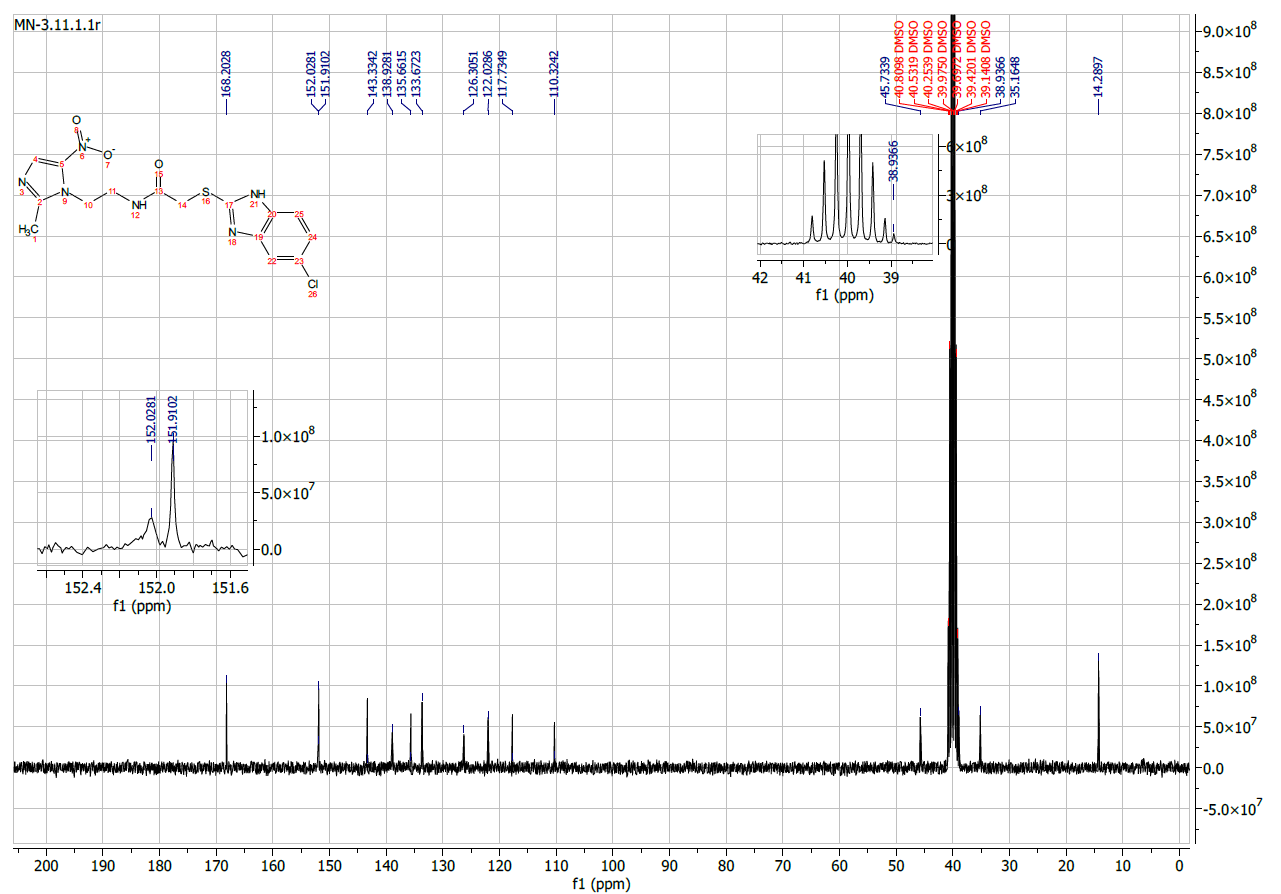


^13^C-NMR spectrum of compound **5c**


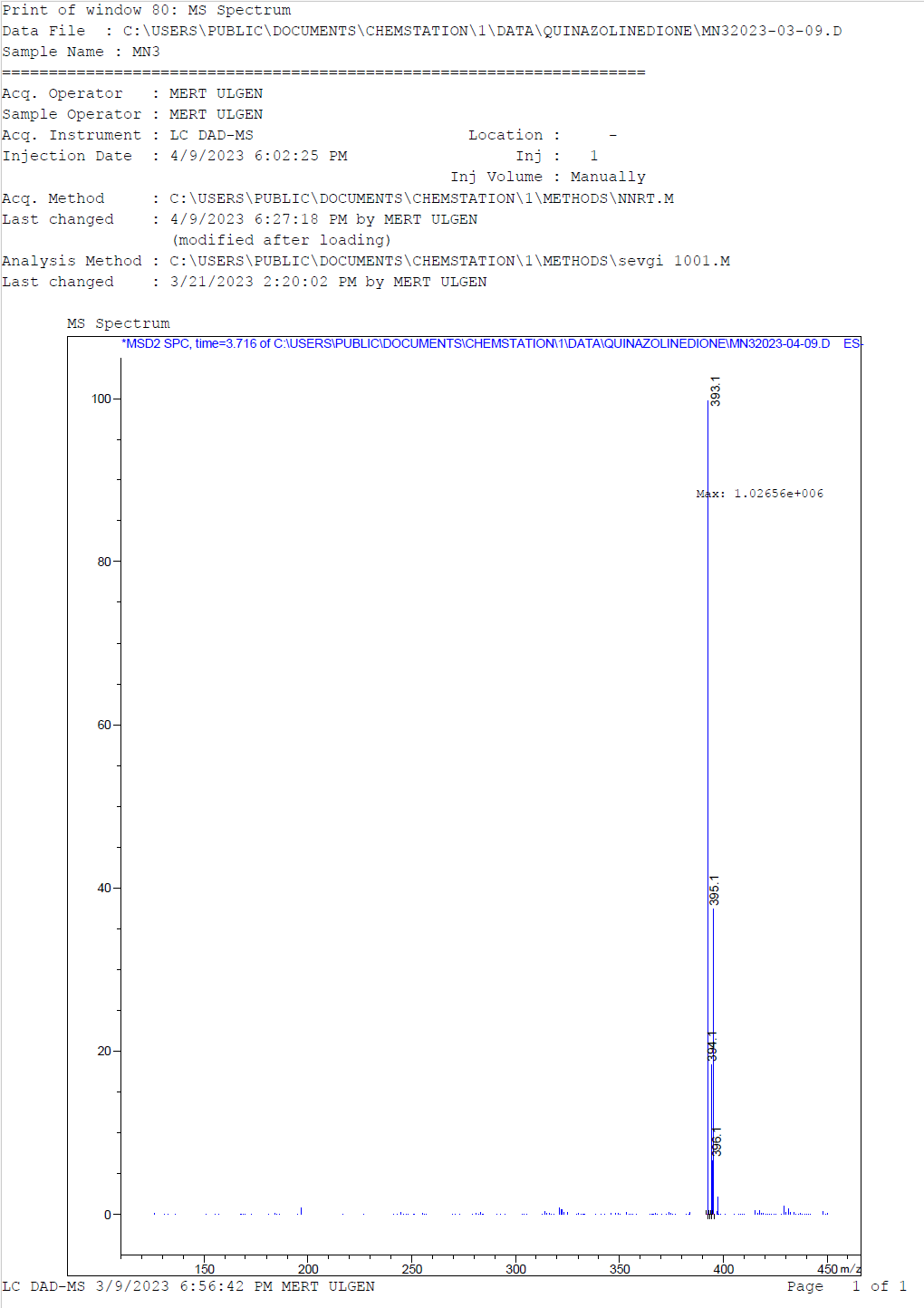


MS spectrum of the compound **5c**


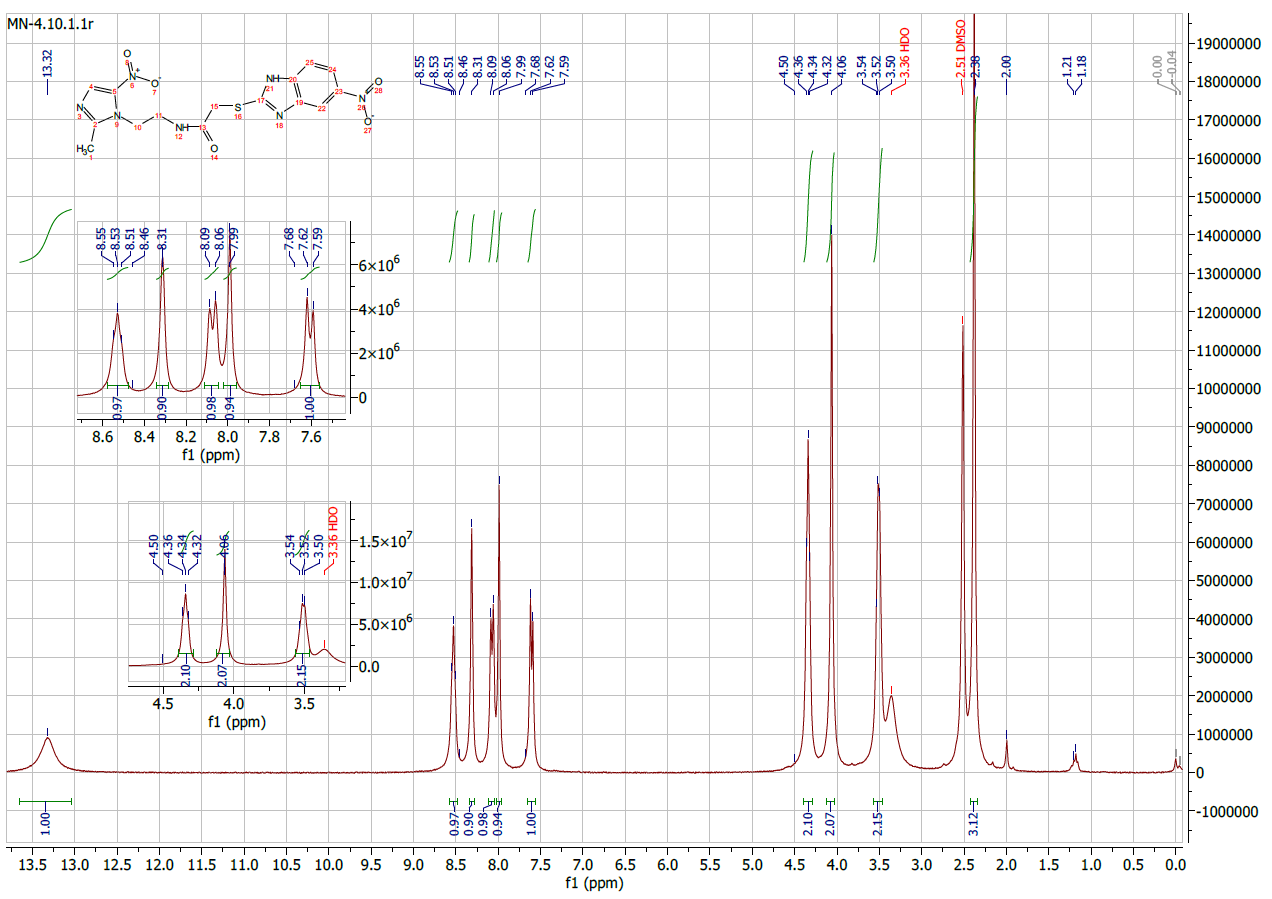


^1^H-NMR spectrum of the compound **5d**


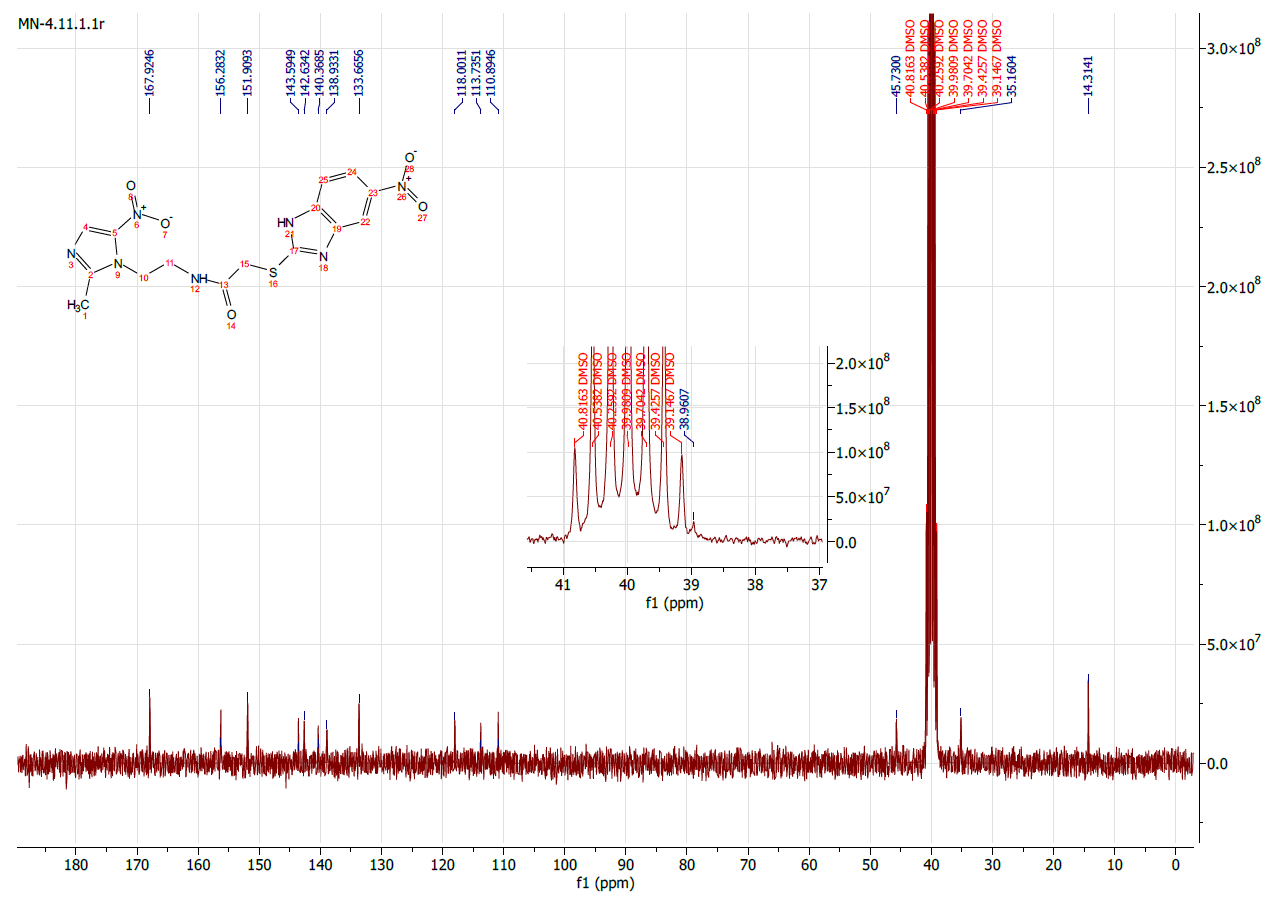


^13^C-NMR spectrum of compound **5d**


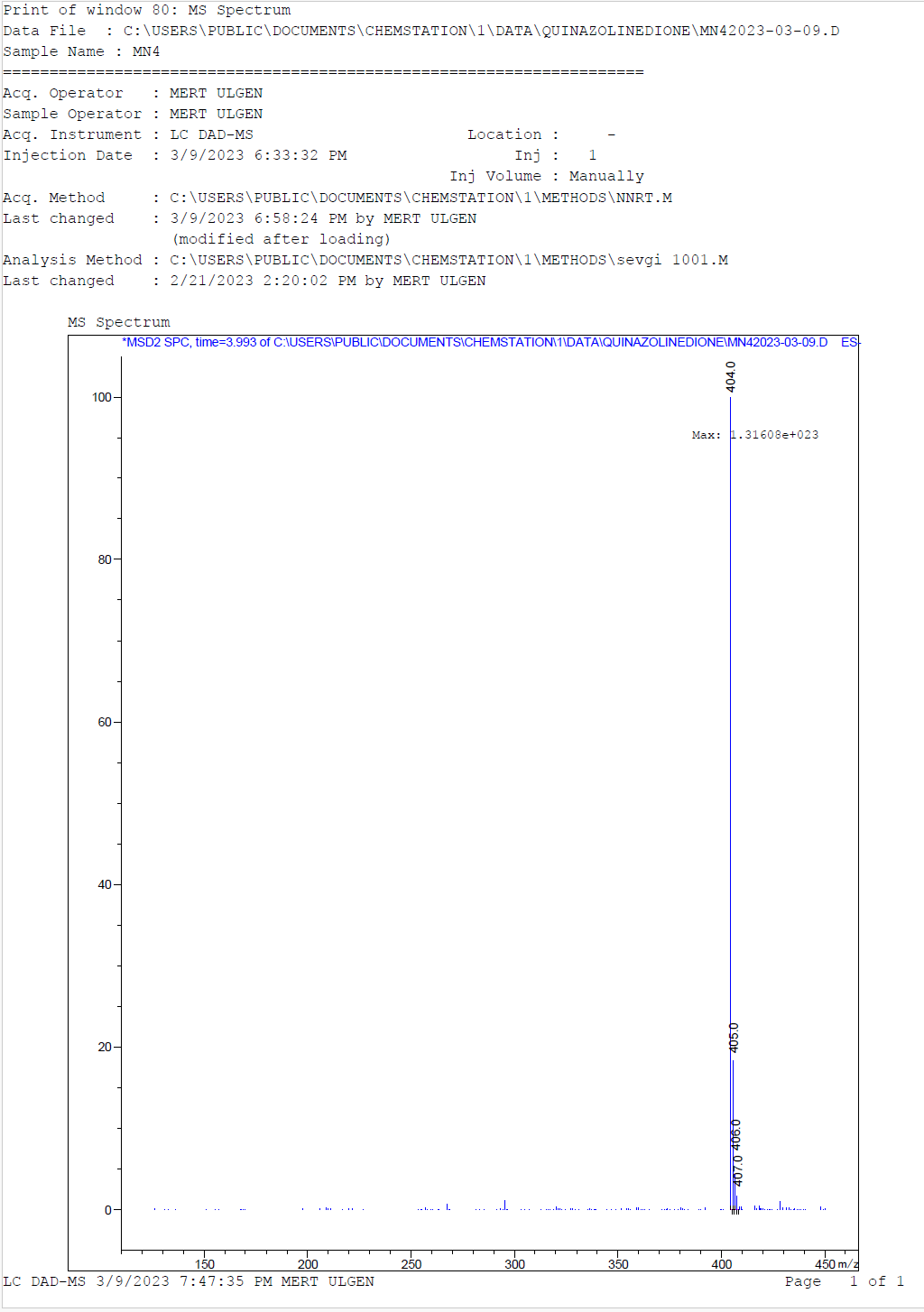


MS spectrum of the compound **5d**


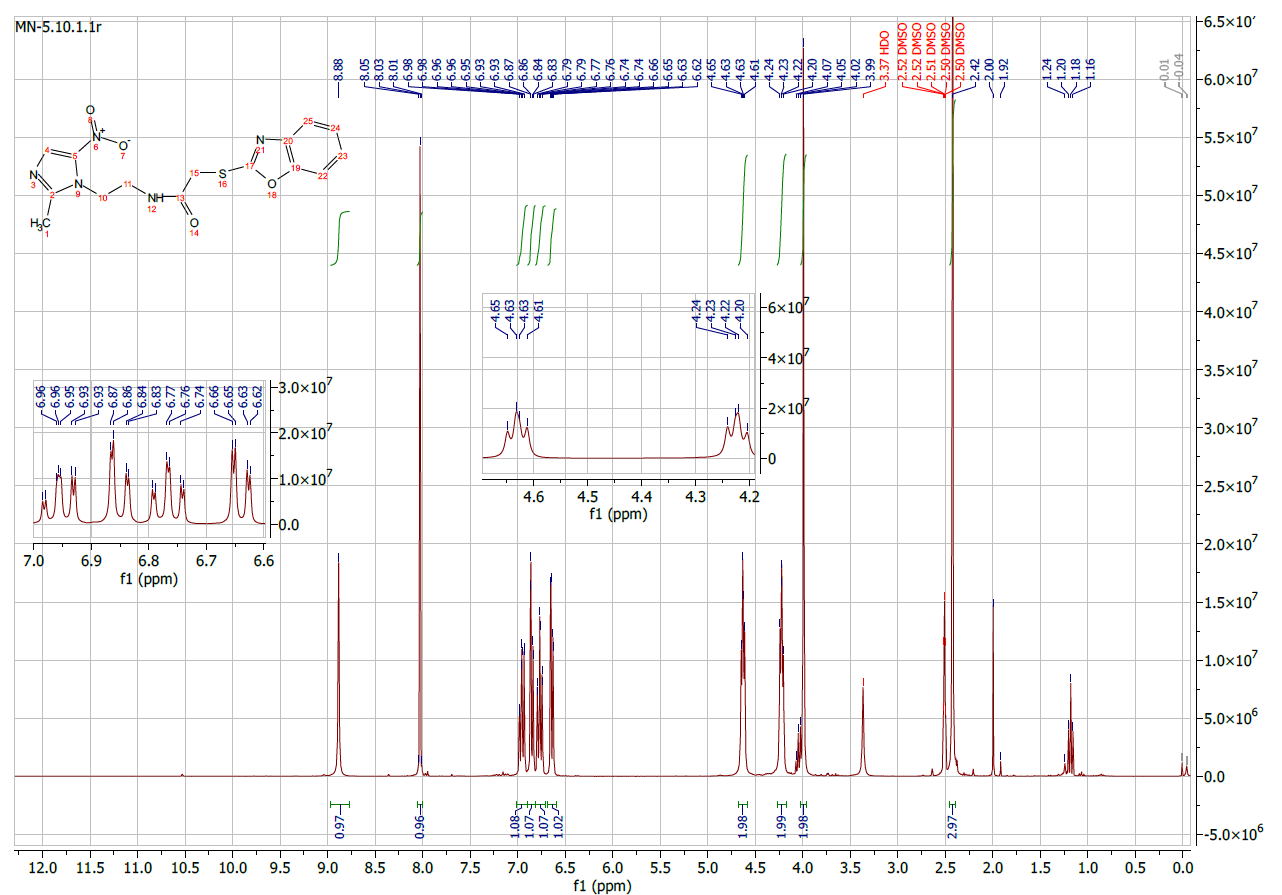


^1^H-NMR spectrum of the compound **5e**


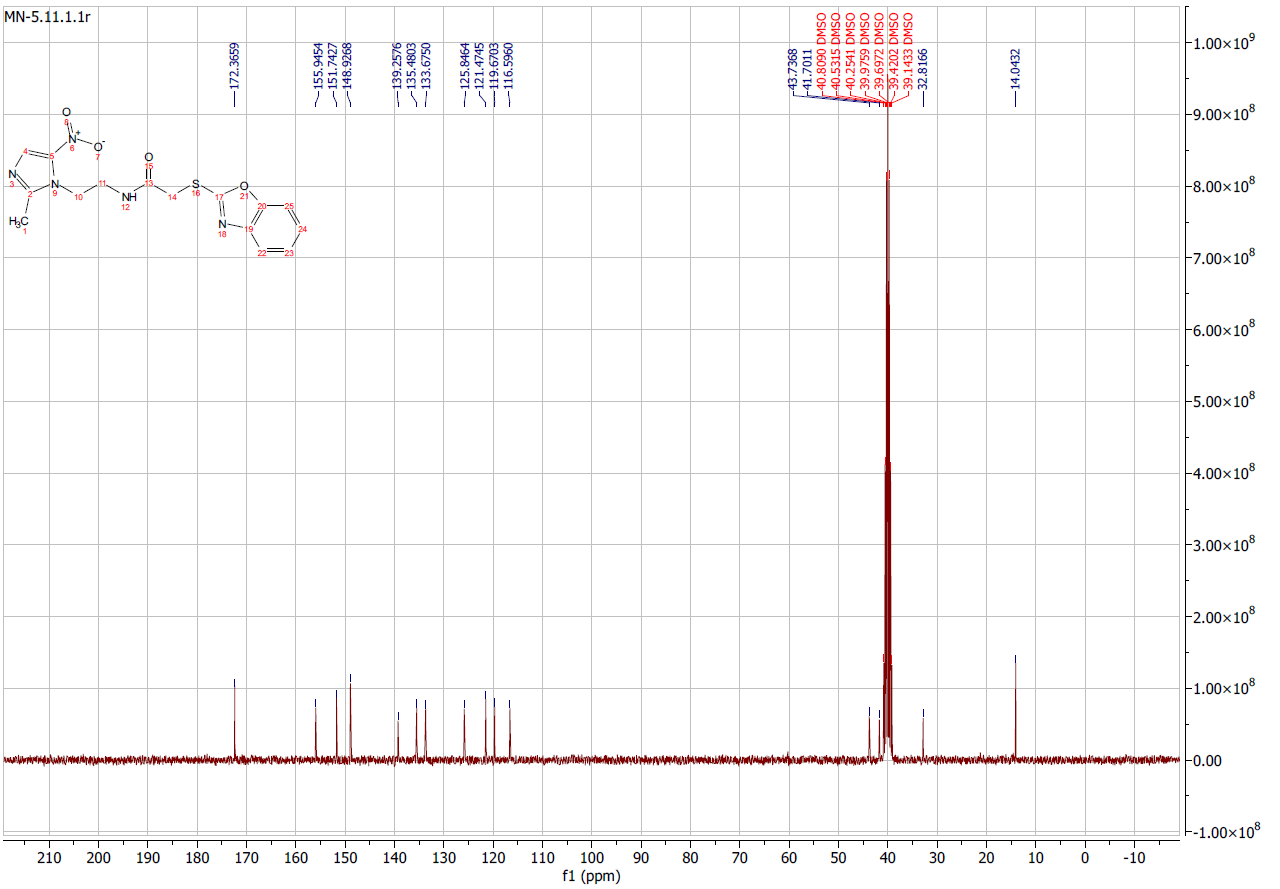


^13^C-NMR spectrum of compound **5e**


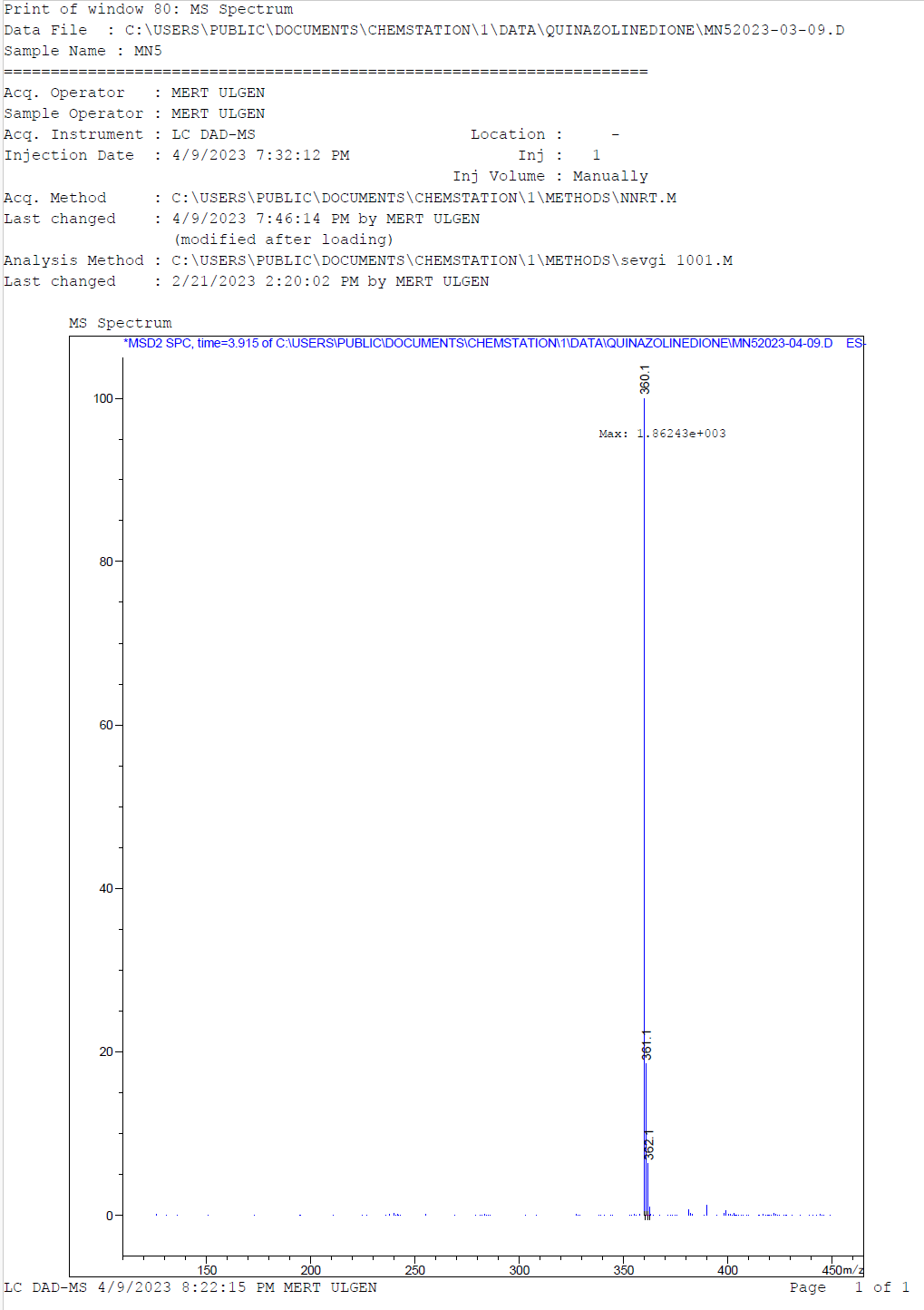


MS spectrum of the compound **5e**


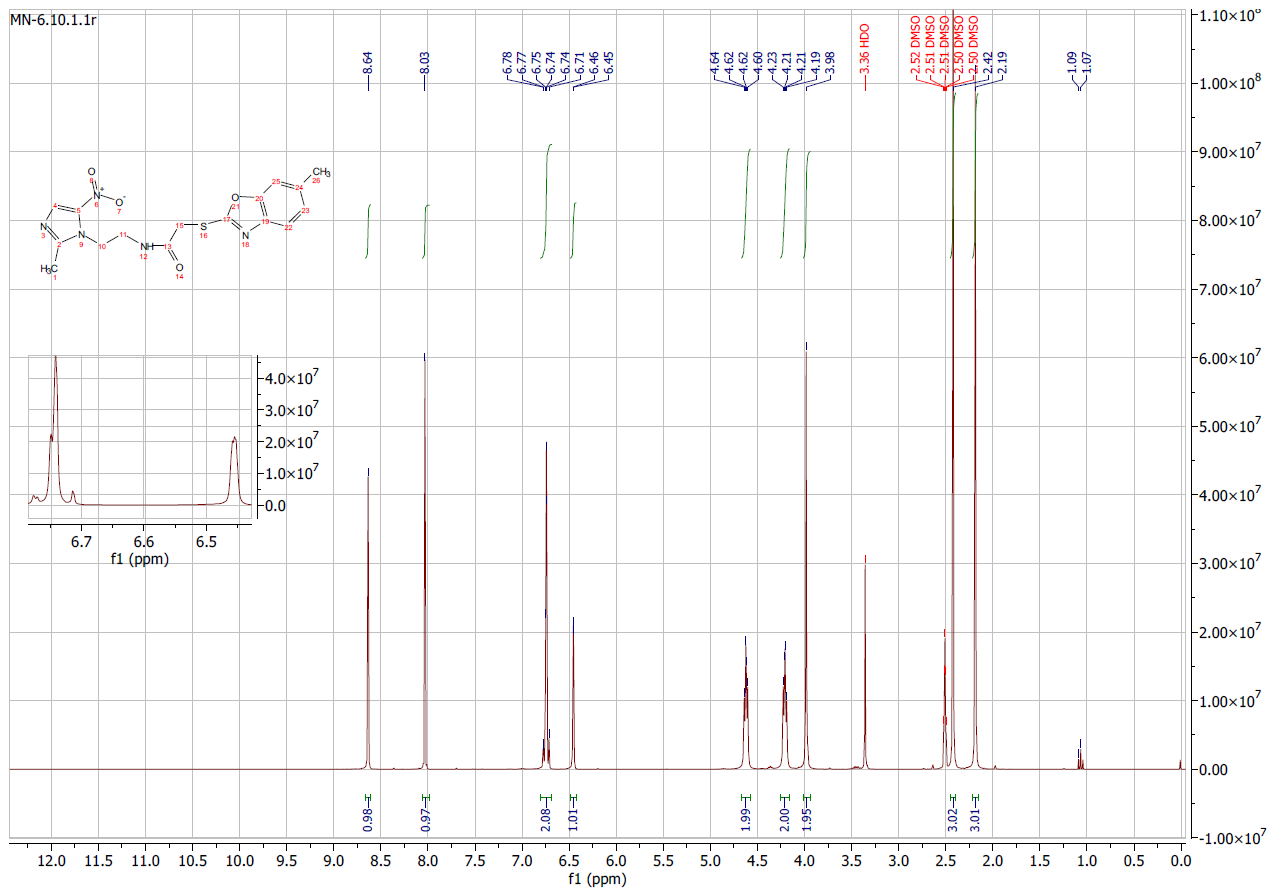


^1^H-NMR spectrum of the compound **5f**


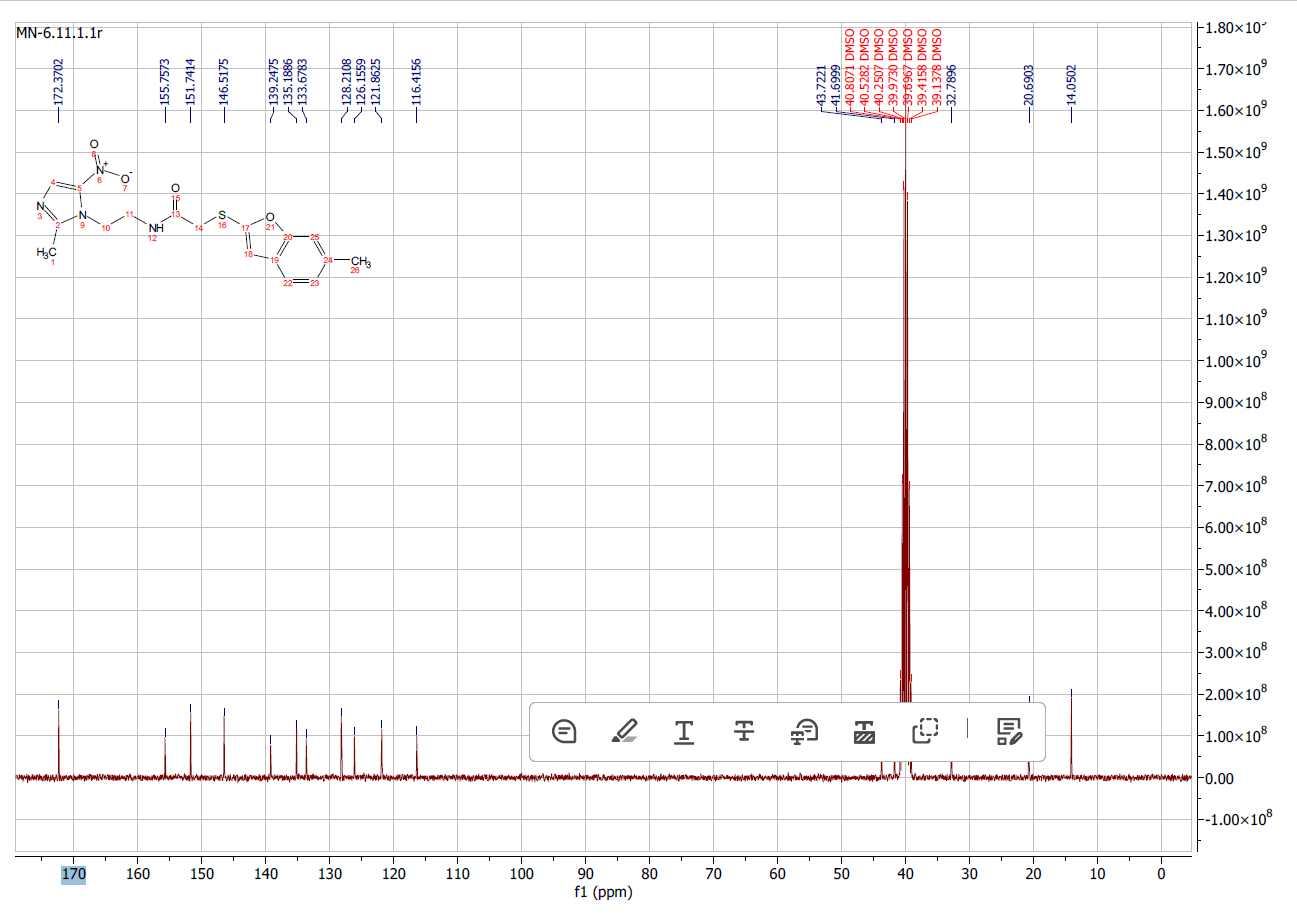


^13^C-NMR spectrum of compound **5f**


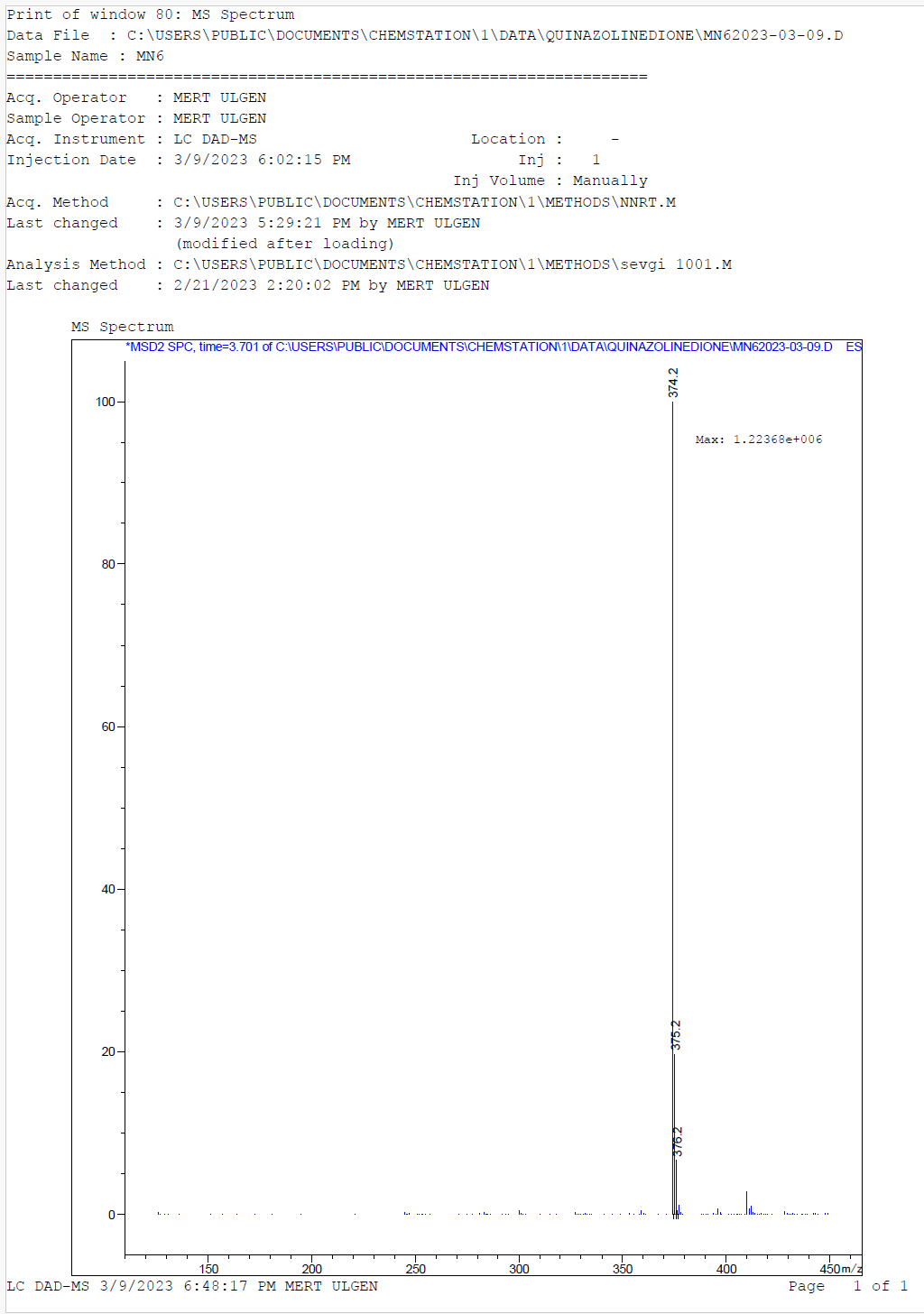


MS spectrum of the compound **5f**


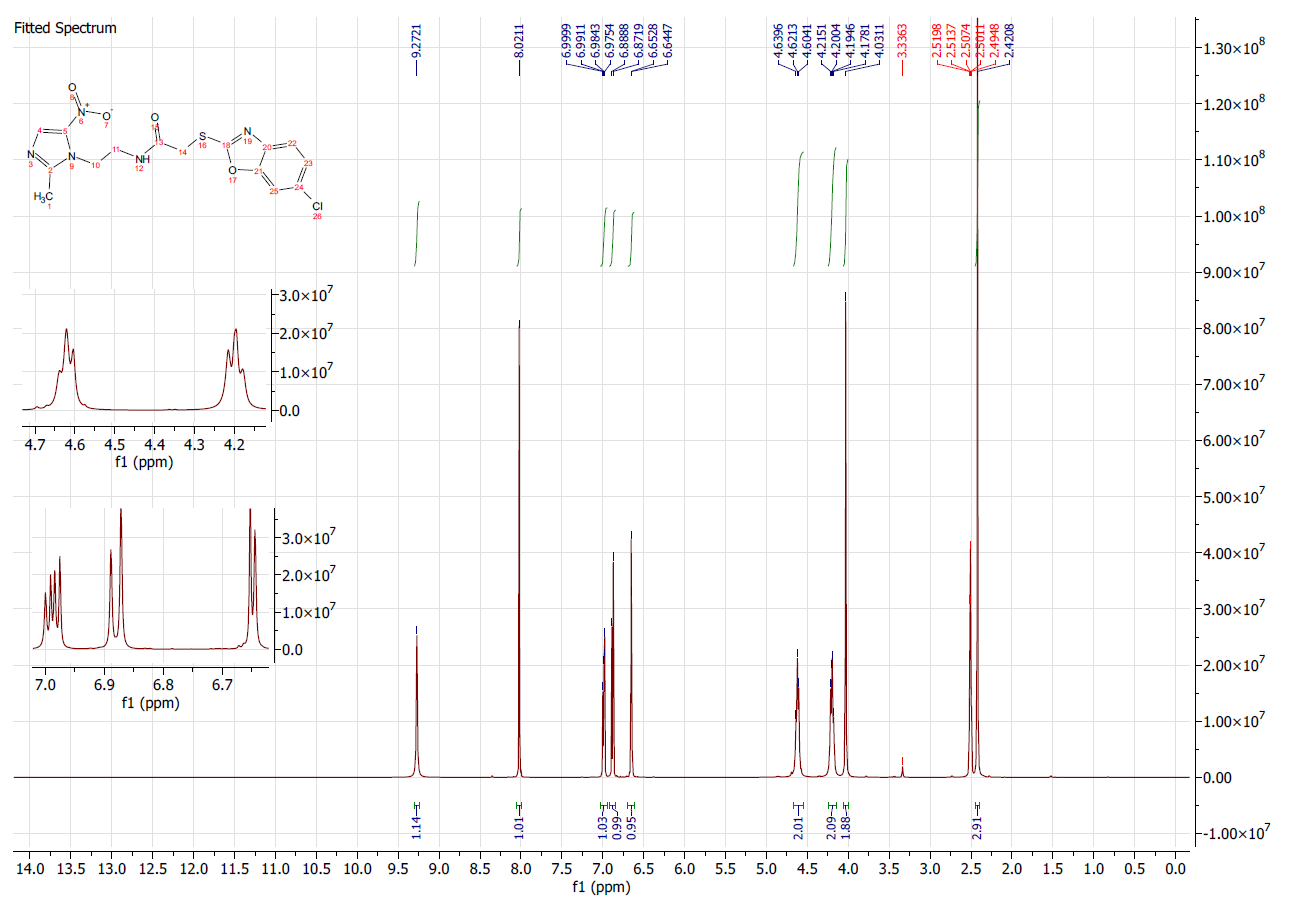


^1^H-NMR spectrum of the compound **5g**


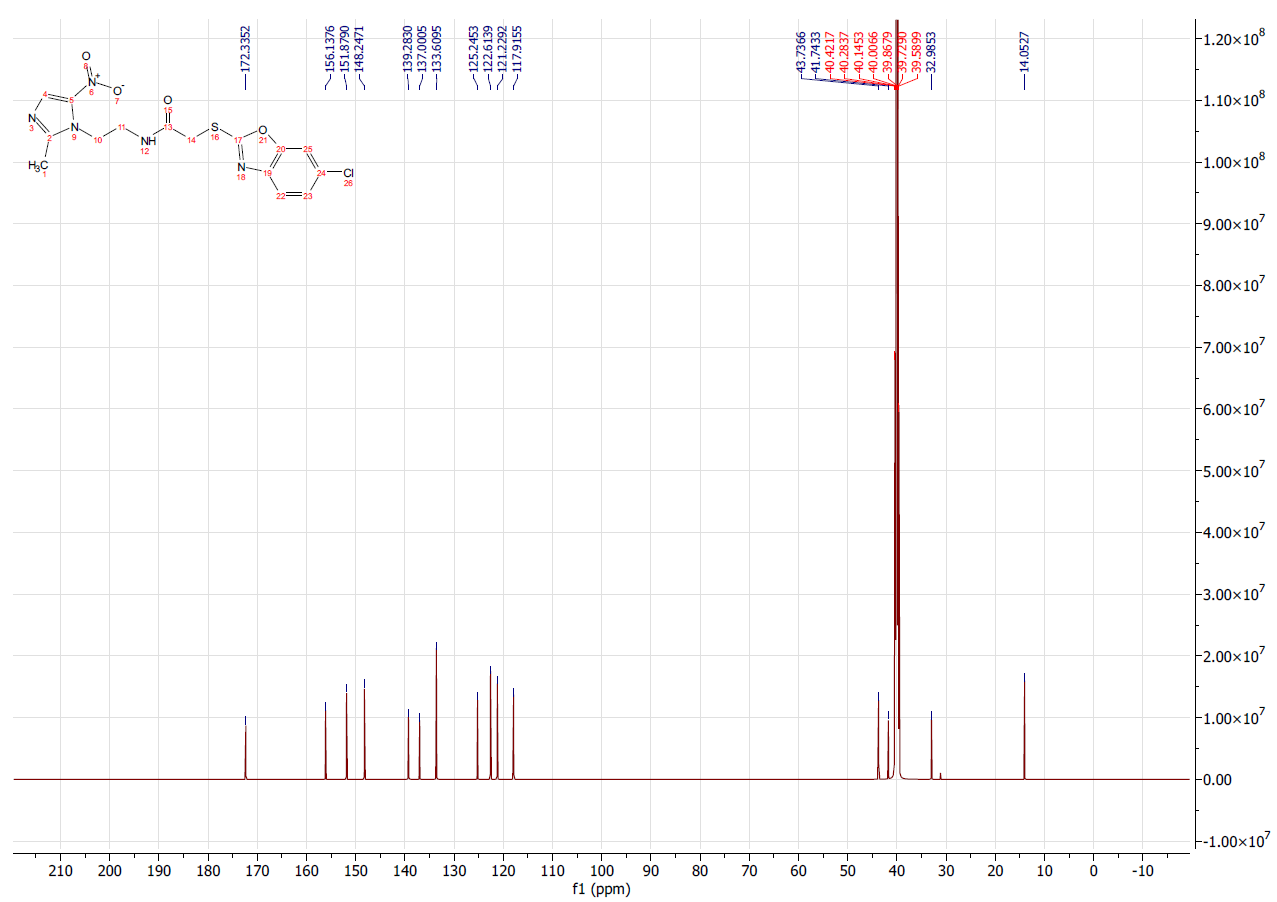


^13^C-NMR spectrum of compound **5g**


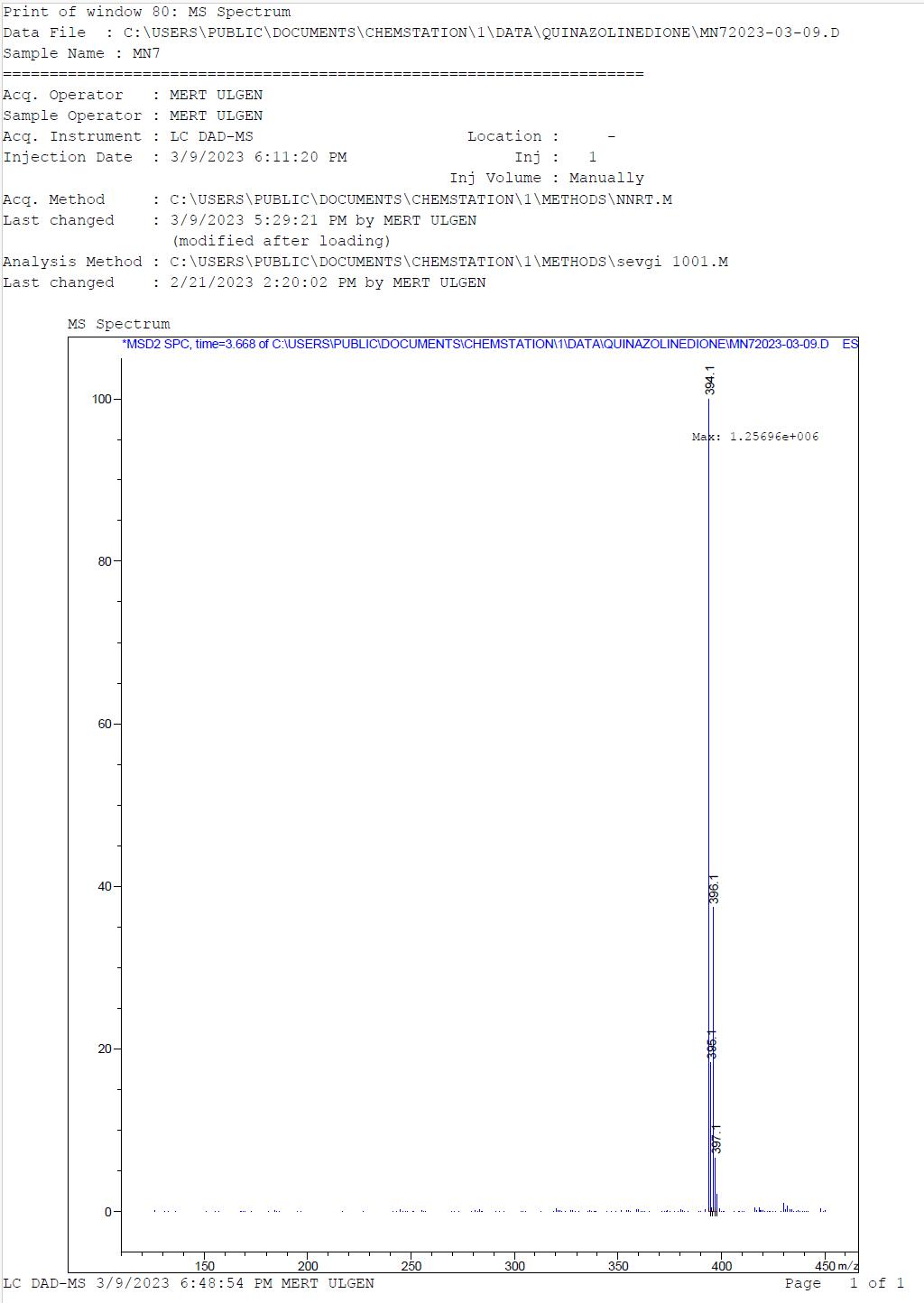


MS spectrum of the compound **5g**


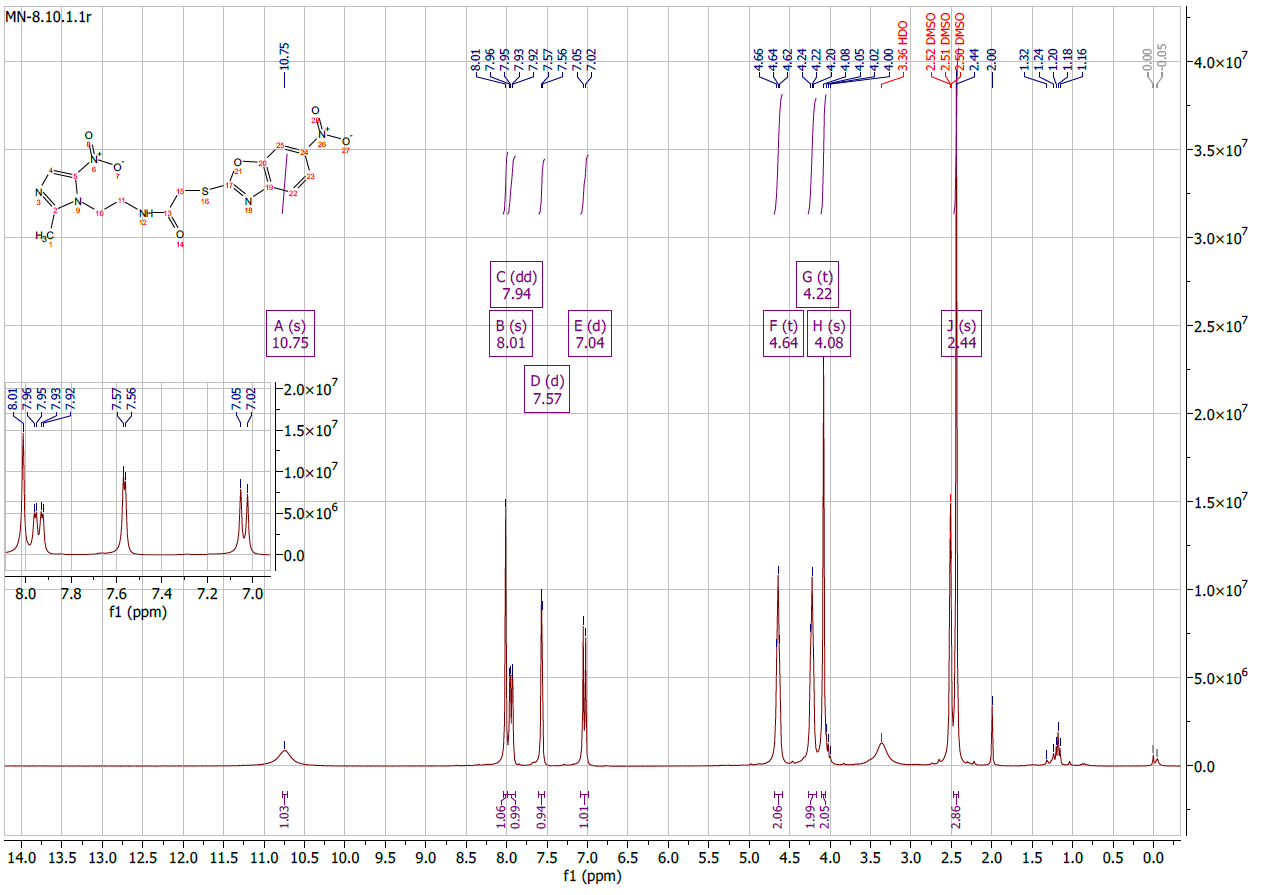


^1^H-NMR spectrum of the compound **5h**


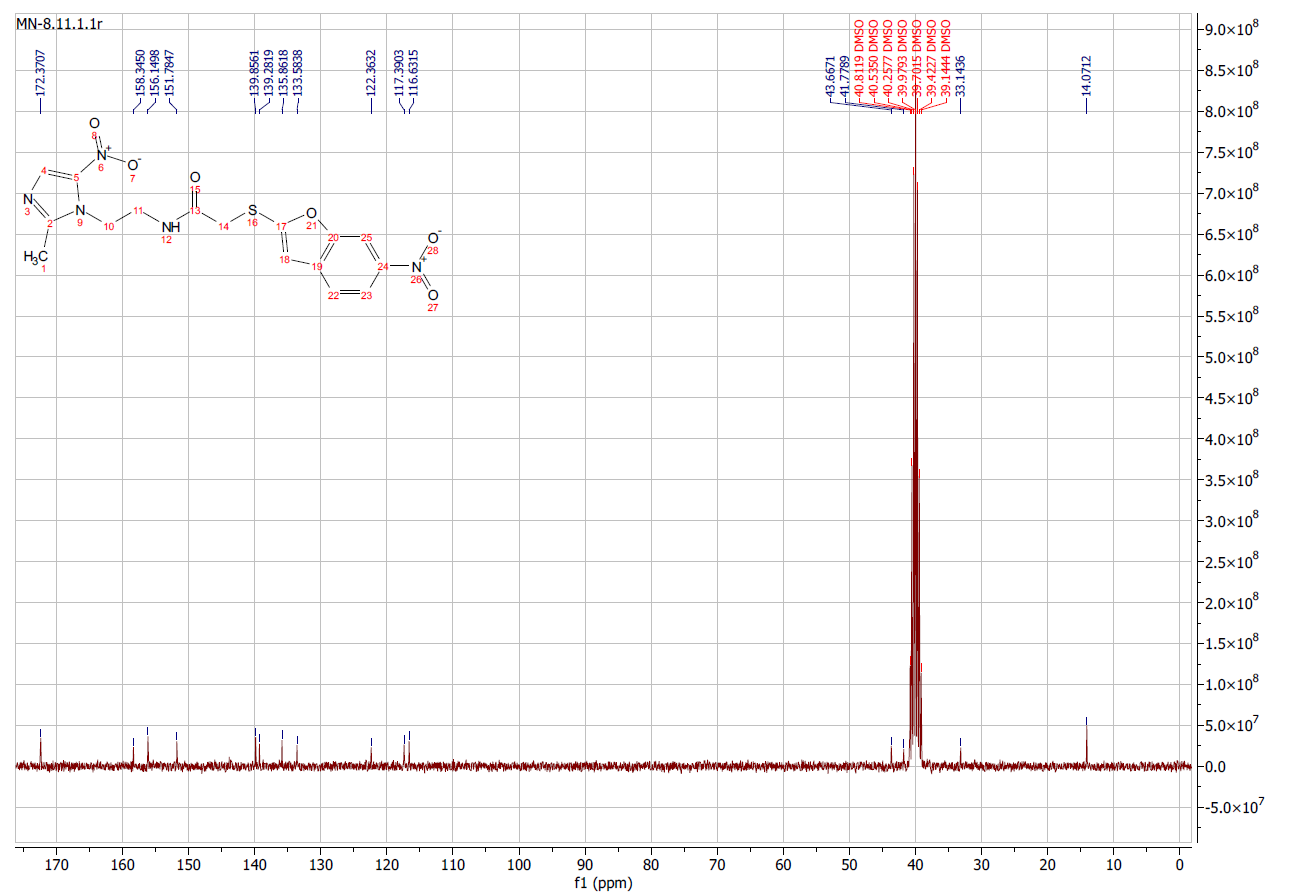


^13^C-NMR spectrum of compound **5h**


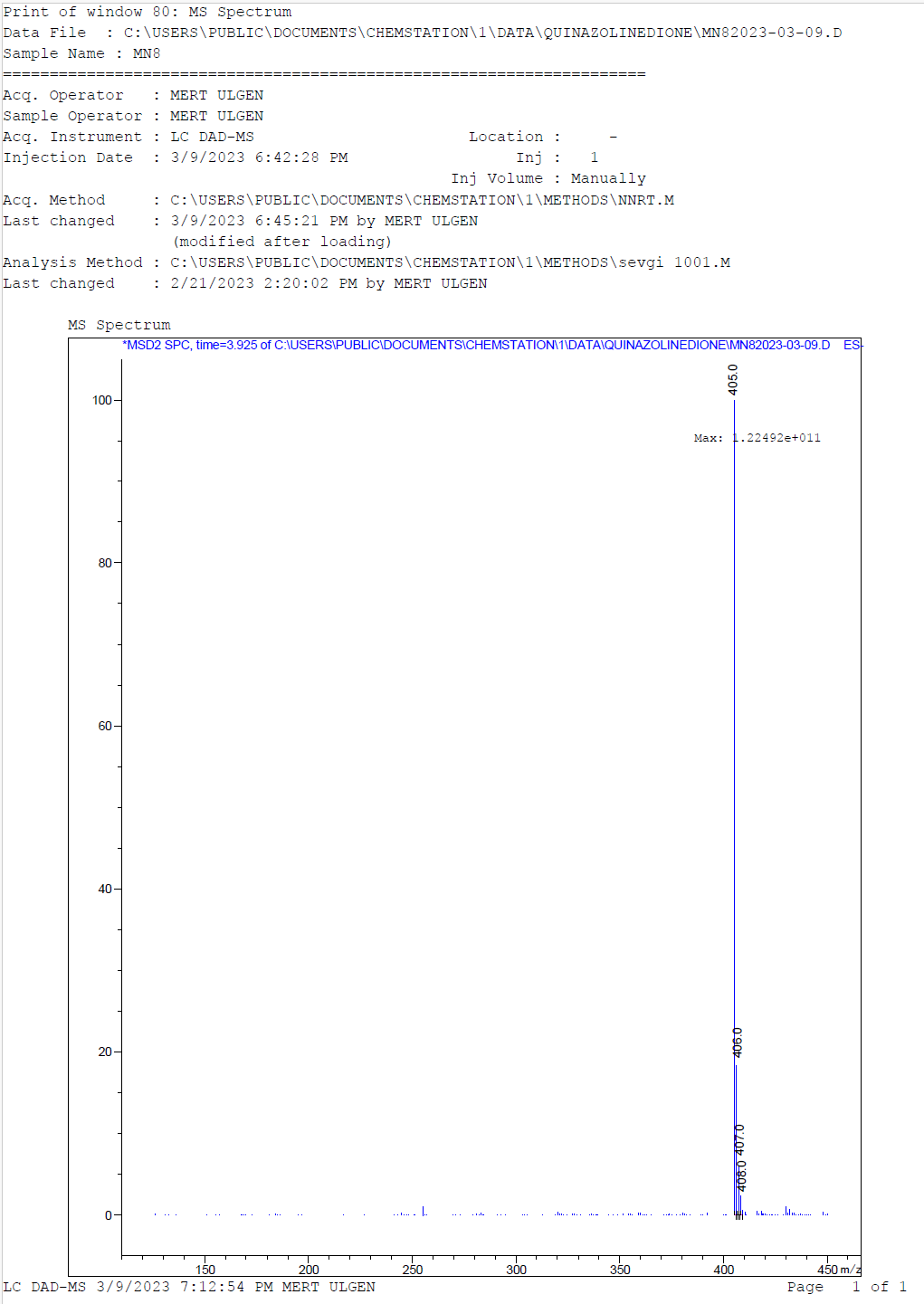


MS spectrum of the compound **5h**


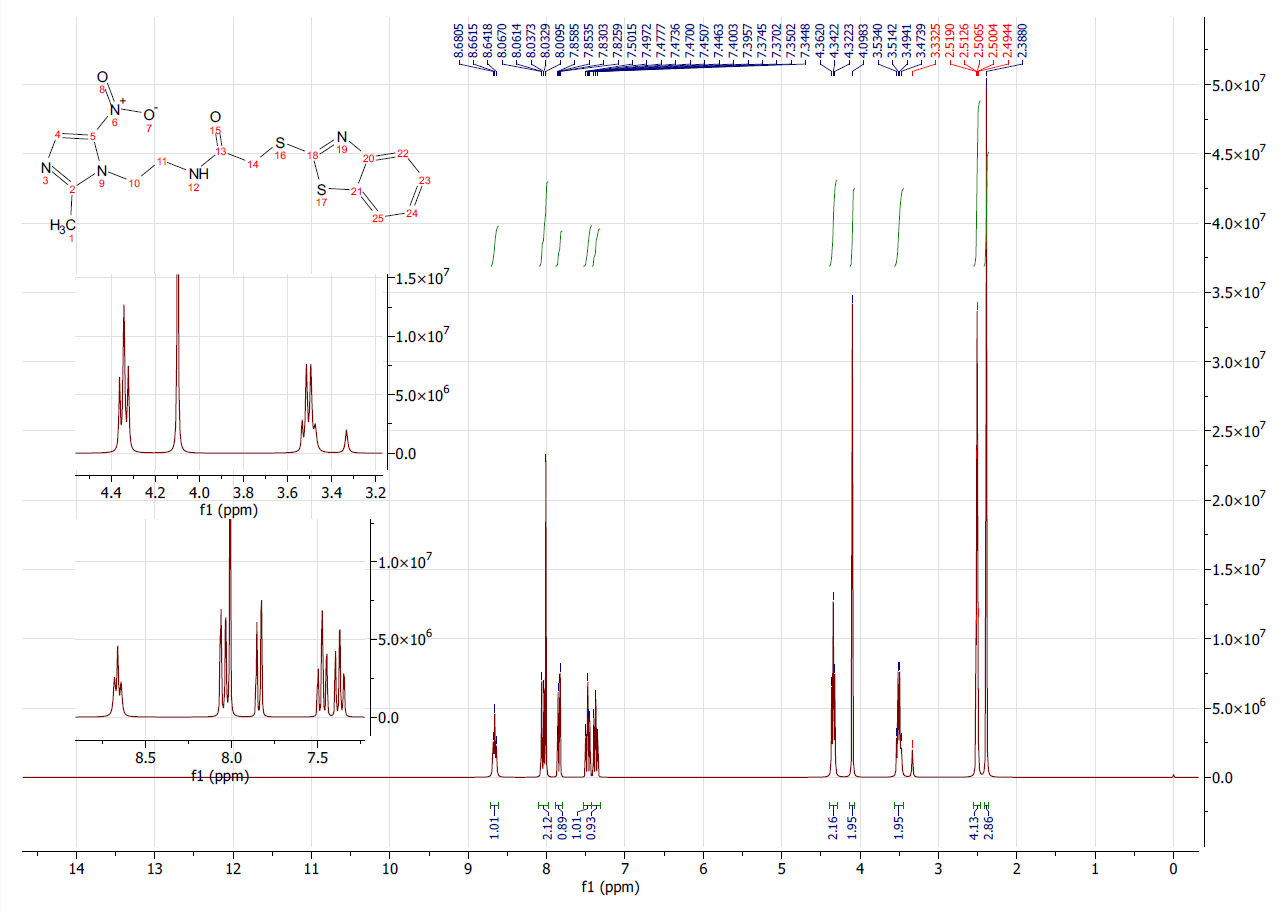


^1^H-NMR spectrum of the compound **5i**


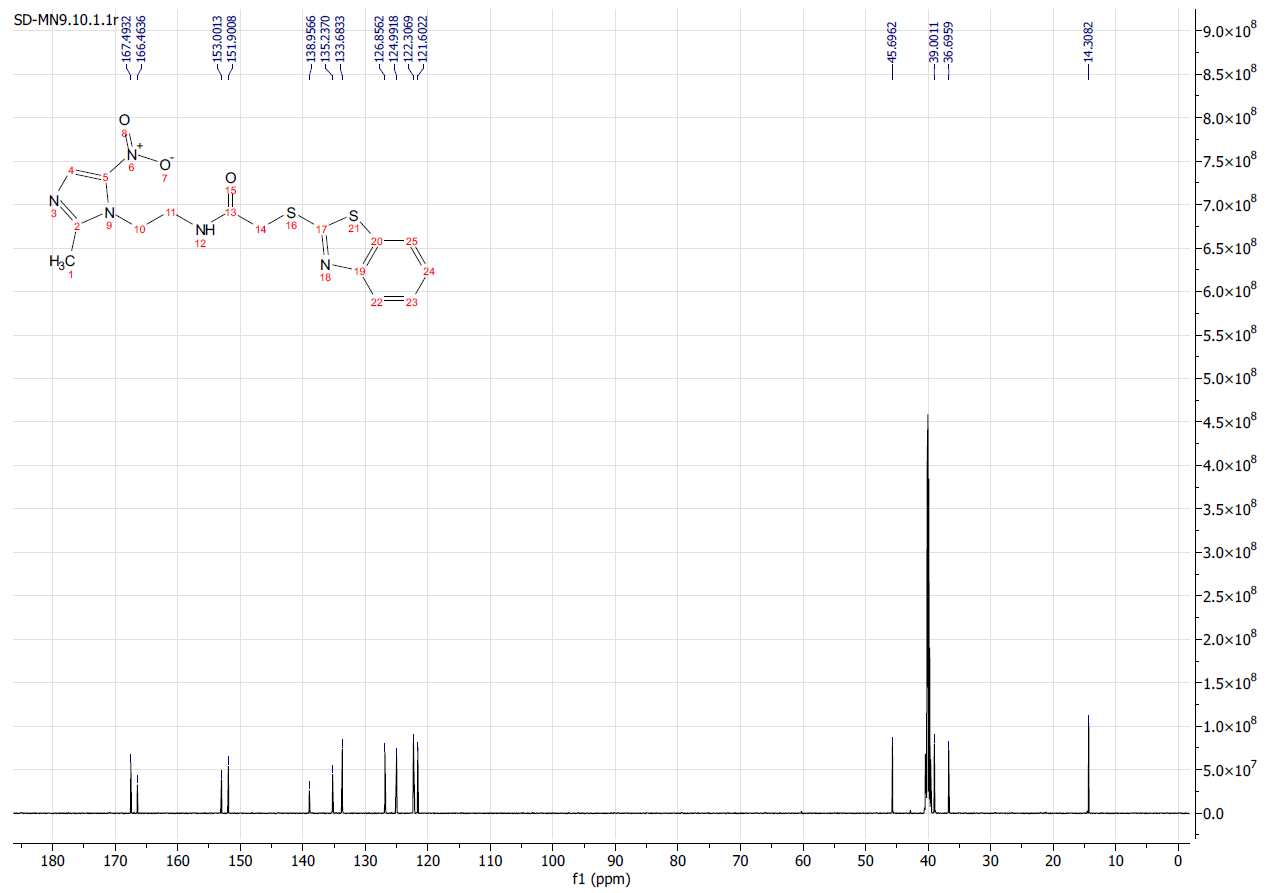


^13^C-NMR spectrum of compound **5i**


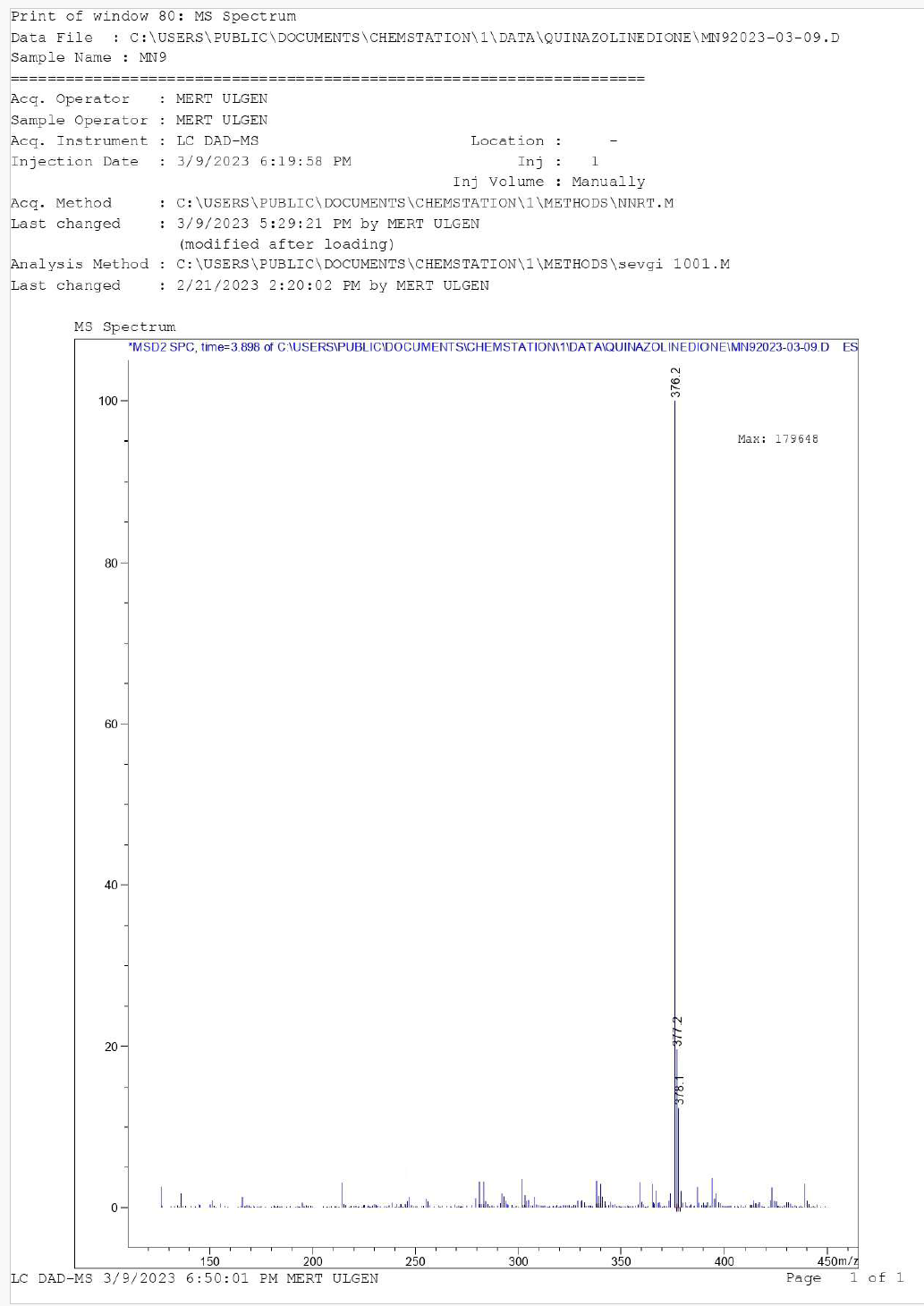


MS spectrum of the compound **5i**


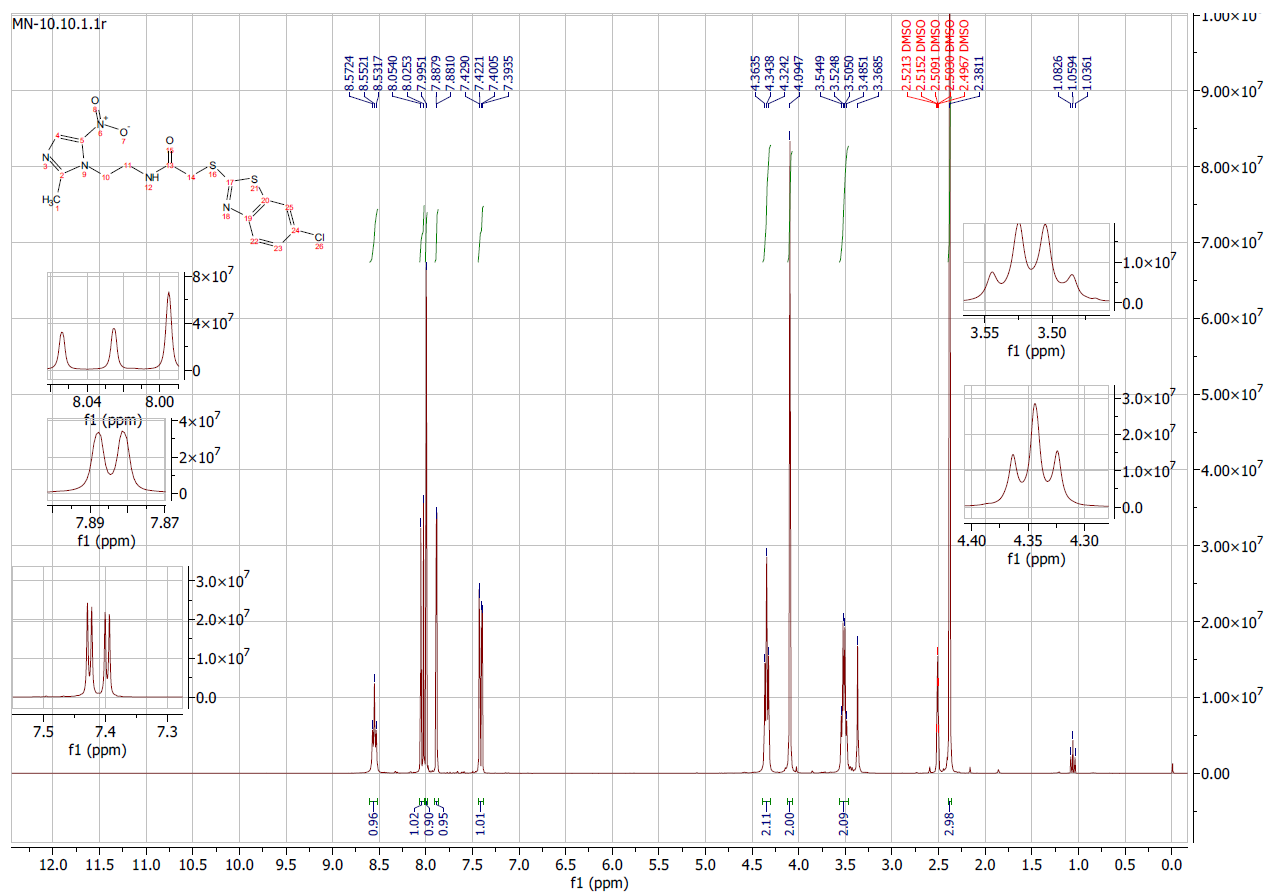


^1^H-NMR spectrum of the compound **5j**


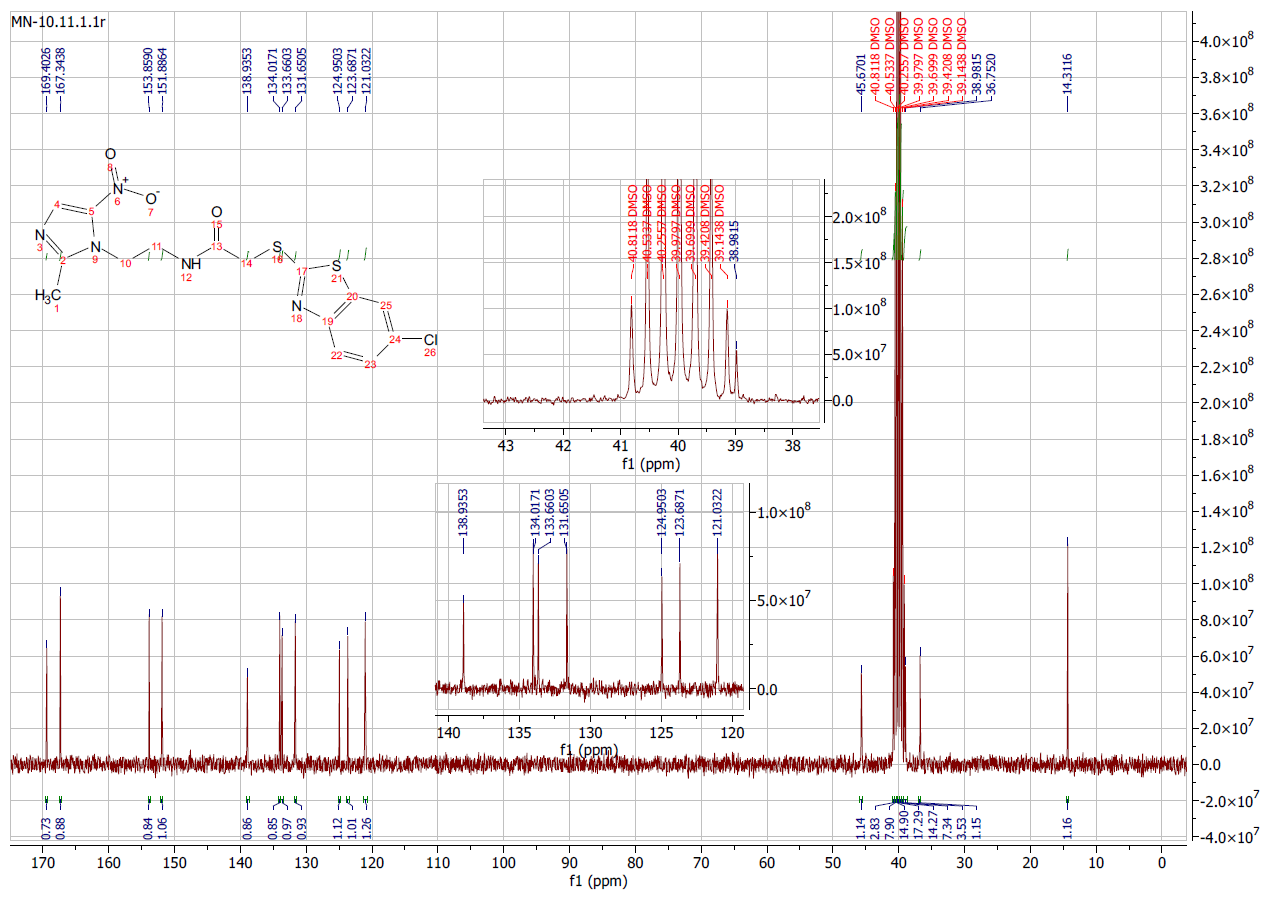


^13^C-NMR spectrum of compound **5j**


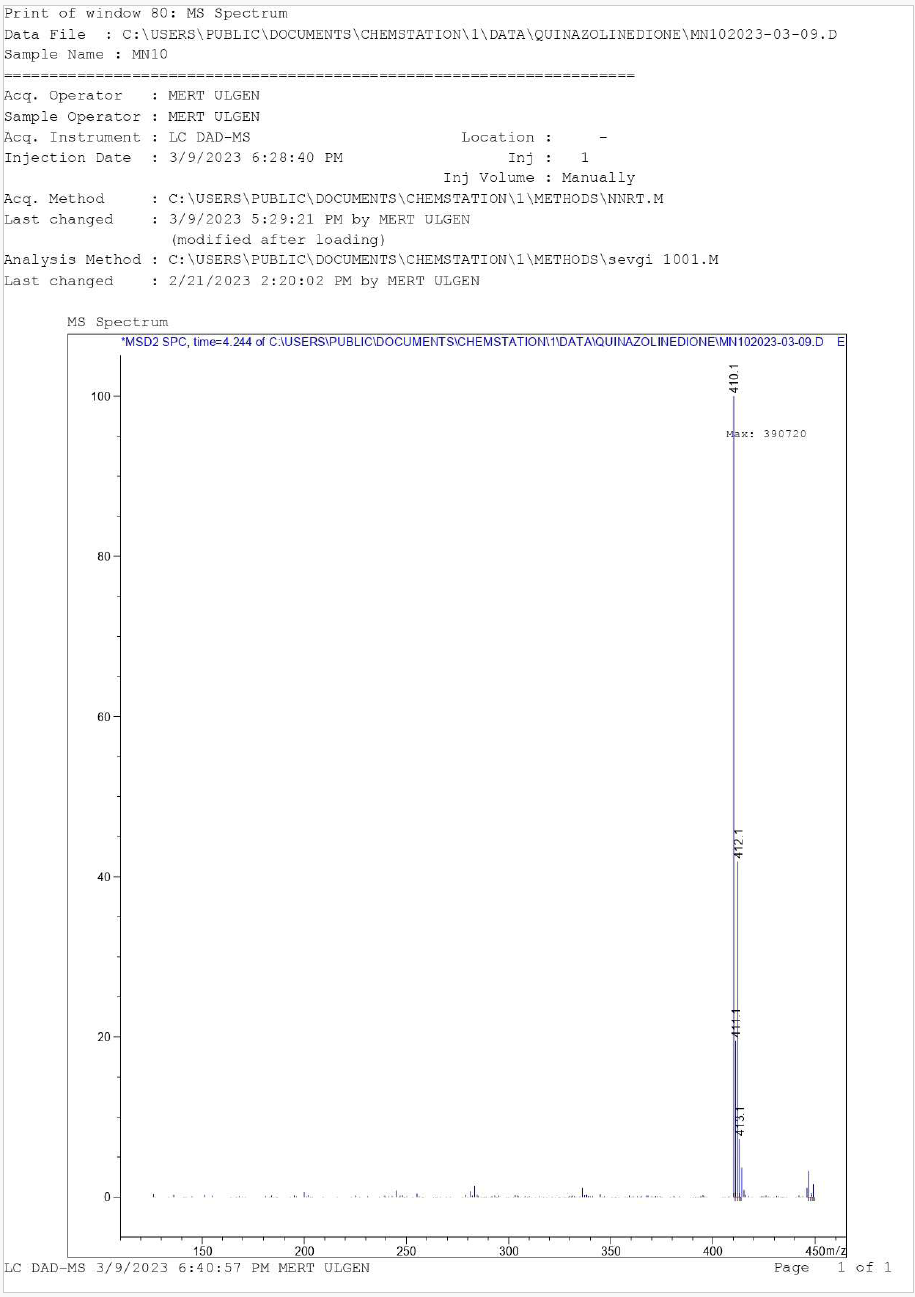


MS spectrum of the compound **5j**
